# Hyperglycemia-induced P300/CBP acetyltransferase drives ZEB2-mediated proinflammatory macrophages and delays wound healing

**DOI:** 10.1172/jci.insight.192146

**Published:** 2026-01-29

**Authors:** Soumyajit Roy, Debarun Patra, Palla Ramprasad, Shivam Sharma, Parul Katiyar, Ashvind Bawa, Kanhaiya Singh, Kulbhushan Tikoo, Suman Dasgupta, Chandan K. Sen, Durba Pal

**Affiliations:** 1 Department of Biomedical Engineering, Indian Institute of Technology Ropar, Rupnagar, Punjab, India.; 2 Stanford Cardiovascular Institute, Stanford University School of Medicine, Stanford, California, USA.; 3 Department of Pharmacology and Toxicology, National Institute of Pharmaceutical Education and Research, S.A.S. Nagar, Punjab, India.; 4 Department of Surgery, McGowan Institute for Regenerative Medicine, University of Pittsburgh, Pittsburgh, Pennsylvania, USA.; 5 Department of General Surgery, Dayanand Medical College & Hospital, Ludhiana, Punjab, India.; 6 Department of Molecular Biology and Biotechnology, Tezpur University, Napaam, Sonitpur, Assam, India.

**Keywords:** Dermatology, Immunology, Inflammation, Diabetes, Epigenetics, Macrophages

## Abstract

Chronic hyperglycemia changes the expression of various transcription factors and mRNA transcripts that impair cellular functionality and delay wound healing. Zinc finger E-box–binding homeobox 2 (ZEB2), a key transcription factor, maintains tissue-specific macrophage identities; however, its role in regulating macrophage polarization during wound healing under hyperglycemic conditions remains unclear. Here, we found that persistent hyperglycemia increases ZEB2 expression in wound macrophages via histone acetylation, contributing to chronic inflammation and delayed wound healing. Exposure to high glucose levels activated P300/CBP, a transcriptional coactivator involved in histone acetylation, which enhanced ZEB2 expression in wound macrophages. The forced expression of ZEB2 shifted macrophage polarity toward a proinflammatory state by upregulating myeloid lineage–directed transcription factors. Conversely, silencing *Zeb2* at the wound site reduced hyperglycemia-induced macrophage inflammation. Topical application of C646, an inhibitor of P300, at the wound edges of streptozotocin-induced high-fat diet–fed diabetic mice significantly decreased ZEB2 expression, reduced inflammation, and accelerated wound healing. Therefore, targeted inhibition of P300 represents a promising therapeutic strategy for improving diabetic wound healing by modulating ZEB2-driven inflammation in wound macrophages.

## Abstract 2

Hyperglycemia-induced ZEB2 expression via P300 drives inflammation, and delays wound healing. Silencing ZEB2 or using the P300 inhibitor C646 reduces inflammation and promotes wound healing.

## Graphical abstract

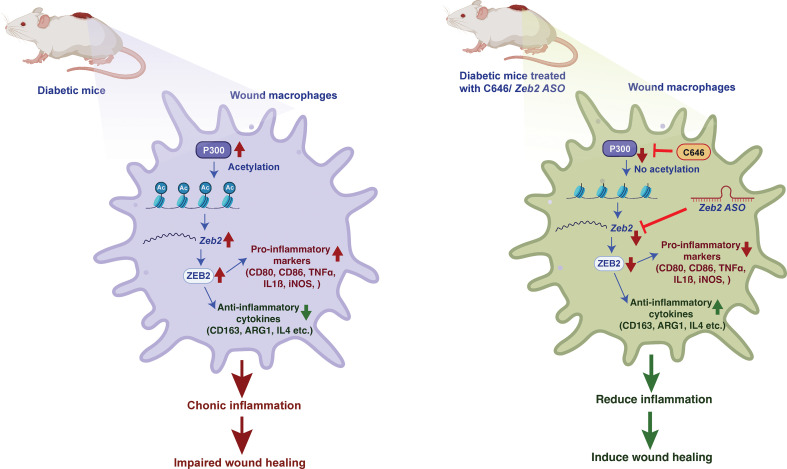

## Introduction

The development of a diabetic foot ulcer (DFU) is a severe and common complication of chronic diabetes, driven by high-glucose-induced vascular and microvascular damage. This damage disrupts peripheral blood flow, reduces oxygen and nutrient levels at the wound site, and stalls the healing process (1). Immune cells play an essential role in managing damaged tissue by releasing cytokines and enzymes to eliminate debris and combat infections (2).

Macrophages play a central role in wound healing, tissue regeneration, and host defense by establishing a sterile and regulated environment. During wound healing, macrophages transition to an antiinflammatory state and secrete growth factors that promote angiogenesis, fibroblast proliferation, and tissue regeneration. Their functions balance pro- and antiinflammatory actions, and any disruption in their activity can hinder the healing process (3).

Hyperglycemia intensifies inflammation during wound healing by repressing the expression of genes crucial for antiinflammatory macrophage polarization while upregulating proinflammatory macrophage polarization. This chronic inflammation is characterized by elevated levels of proinflammatory cytokines and chemokines such as TNF-α, IL-1β, and IL-6. Additionally, low oxygen tension (hypoxia) activates NF-κB signaling pathways, amplifies proinflammatory responses, and triggers the hypoxia-inducible factor 1-α (HIF1α)/zinc finger E-box–binding homeobox 2 (ZEB2) axis. ZEB2, also known as SIP1 or ZFXH1B, is a transcription factor highly expressed in macrophages and plays pivotal role in macrophage lineage specification (4). Moreover, ZEB2 is also crucial in epithelial-mesenchymal transition and influences granulocyte macrophage progenitor differentiation (5).

Recent findings highlight the role of histone tail acetylation in metabolic and mitogenic insulin signaling regulation (6). Hyperglycemia-induced dysregulation of histone acetyltransferases (HATs) and deacetylases significantly contributes to the development of diabetic retinopathy (7). Additionally, diabetes-associated advanced glycation end products (AGEs) and reactive oxygen species (ROS) activate NF-κB and increase HAT activity, all of which act as major transcriptional coactivators enhancing inflammatory cytokine production (8). Furthermore, reducing the activity of receptors for AGEs (RAGE) by methylglyoxal treatment decreases NF-κB p65 acetylation and suppresses TNF-α production (9).

The present study reveals a role for ZEB2 in sustaining the prolonged proinflammatory state of macrophages in diabetic wounds by increasing histone acetylation. Our findings uncovered that pharmacological inhibition of acetyltransferase P300 significantly reduced ZEB2 protein levels, rescuing macrophages’ proinflammatory state and alleviating diabetic wound healing.

## Results

### Hyperglycemia-induced ZEB2 expression drives proinflammatory responses in wound macrophages.

**Figure 1:**
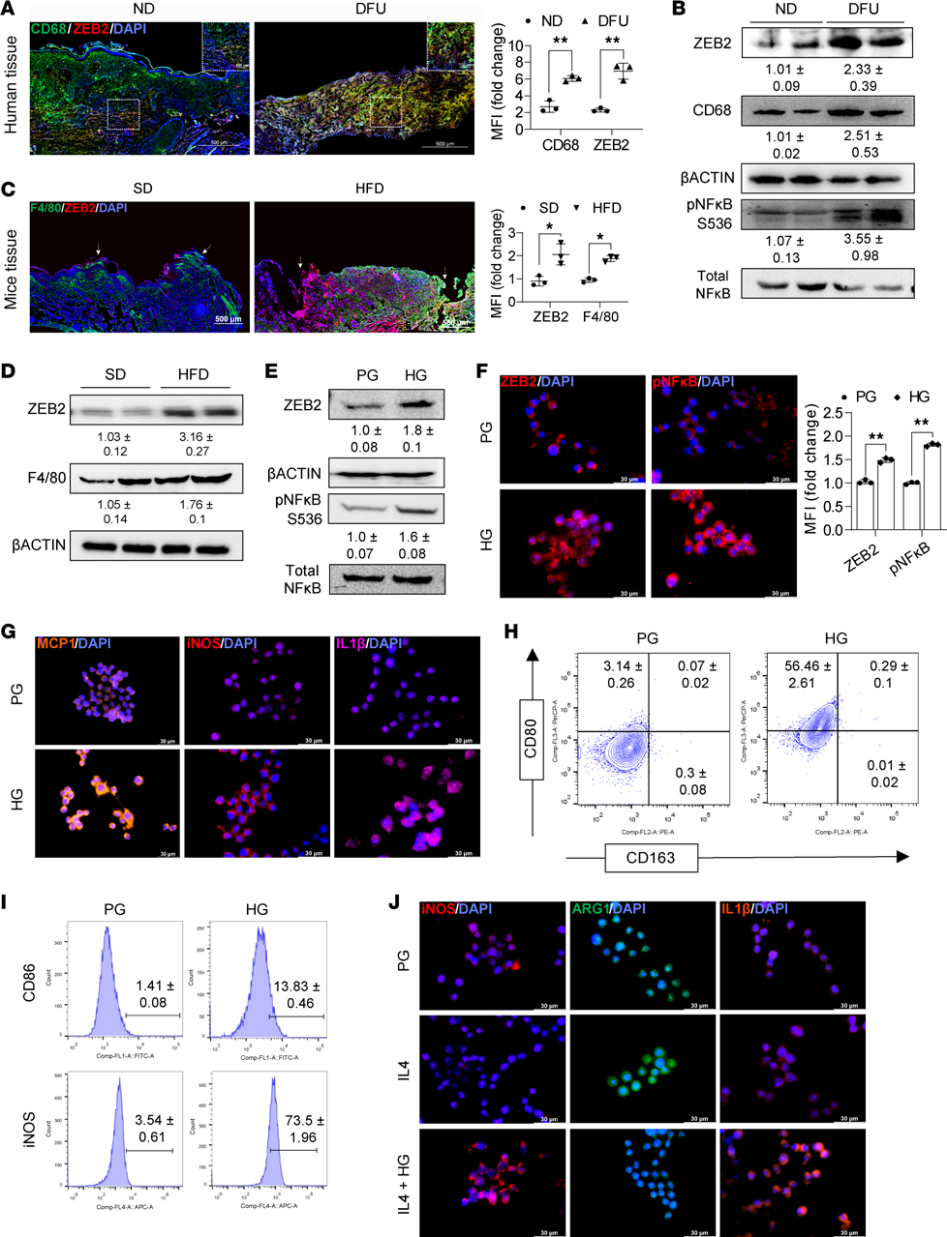
Elevated glucose levels induce the expression of ZEB2 and activate proinflammatory pathways within macrophage populations. Protein expression was assessed by (**A**) immunostaining for ZEB2 (red) and CD68 (green) (scale bars: 100 μm [higher magnification] and 500 μm [lower magnification]; *n* = 3/group) and (**B**) Western blot analysis of ZEB2, CD68, and p-NF-κB proteins (*n* = 4/group) in human wound tissue. **P < 0.01 by paired, 2-tailed Student’s *t* test. Expression of ZEB2 and F4/80 were measured by (**C**) immunostaining (scale bars: 500 μm, *n* = 3/group) and (**D**) Western blot analysis (*n* = 4/group) in SD-fed and HFD-fed wound tissue from mice on day 7 (d7). *P < 0.05 by paired, 2-tailed Student’s *t* test. Effect of HG (25 mM D-glucose) induction on ZEB2 and p-NF-κB was examined by (**E**) Western blot and (**F**) immunostaining (scale bars: 30 μm). **P < 0.01 by paired, 2-tailed Student’s *t* test (*n* = 3). Inflammatory status of HG-treated RAW264.7 cells was assessed by (**G**) immunofluorescence analysis of ARG1 and iNOS (scale bars: 30 μm, *n* = 3) and (**H** and **I**) flow cytometric analysis of macrophage surface markers (*n* = 3). (**J**) Representative images of immunostaining for iNOS, ARG1, and IL-1β in PG-treated, IL-4–treated (20 ng/mL for 24 hours), and IL-4 + HG–treated RAW264.7 cells (scale bars: 30 μm; *n* = 3). Experiments were repeated 3 times independently. Data are expressed as mean ± standard deviation. ND, nondiabetic; DFU, diabetic foot ulcer; SD, standard diet; HFD, high-fat diet; PG, physiological glucose level; HG, hyperglycemia.

Elevated glucose levels in the circulation exacerbate its deleterious effects on cells, tissues, and organ systems, causing myriad pathophysiological changes and leading to delayed wound healing in chronically obese diabetic patients (10, 11). Diabetic wounds are characterized by an inflammatory phase where macrophages fail to transition from a proinflammatory to a reparative phenotype (12). In this context, ZEB2 plays a pivotal role, leveraging its diverse functions as a transcription factor to shape and regulate macrophage development and functionality (5). In this study, we observed that, diabetic patients with elevated blood glucose levels (Supplemental Figure 1A; supplemental material available online with this article; https://doi.org/10.1172/jci.insight.192146DS1) exhibited high ZEB2 expression in wound samples, as compared with the nondiabetic patients’ wound samples (Figure 1A). This was accompanied by an expansion of a proinflammatory macrophage population (Supplemental Figure 1B), as indicated by elevated levels of proinflammatory markers *CD80* and *CD68*, and reduced levels of antiinflammatory marker *CD206*, representing an inflammatory milieu at the diabetic wound site. Histological examination revealed reduced granulation tissue, culminating in the lack of an epithelial covering, along with a substantial inflammatory infiltration in diabetic wound samples (Supplemental Figure 1C). This observation suggests a notable impairment in reepithelialization and wound closure in diabetic patients. Additionally, we observed significant upregulation of ZEB2 along with NF-κB activation, a key molecule in inflammatory signaling cascades, in the CD68^+^ wound macrophages from patients with DFU compared with those from nondiabetic participants (Figure 1B). We developed a mouse model of type 2 diabetes by administering streptozotocin (STZ) to C57BL/6J mice fed with a high-fat diet (HFD) to better recapitulate the pathophysiological features of type 2 diabetes, which is more clinically relevant to impaired wound healing, and subsequently generated a rostral excisional wound. STZ-treated, HFD-fed obese mice exhibited significantly higher blood glucose levels compared with vehicle-treated, standard diet–fed (SD-fed) mice (Supplemental Figure 1D). On day 7 (d7) after wounding, there was a marked infiltration of macrophages (Figure 1C), which coincided with an increase in ZEB2 expression in the wound tissue of diabetic mice (Figure 1D).

**Figure 2:**
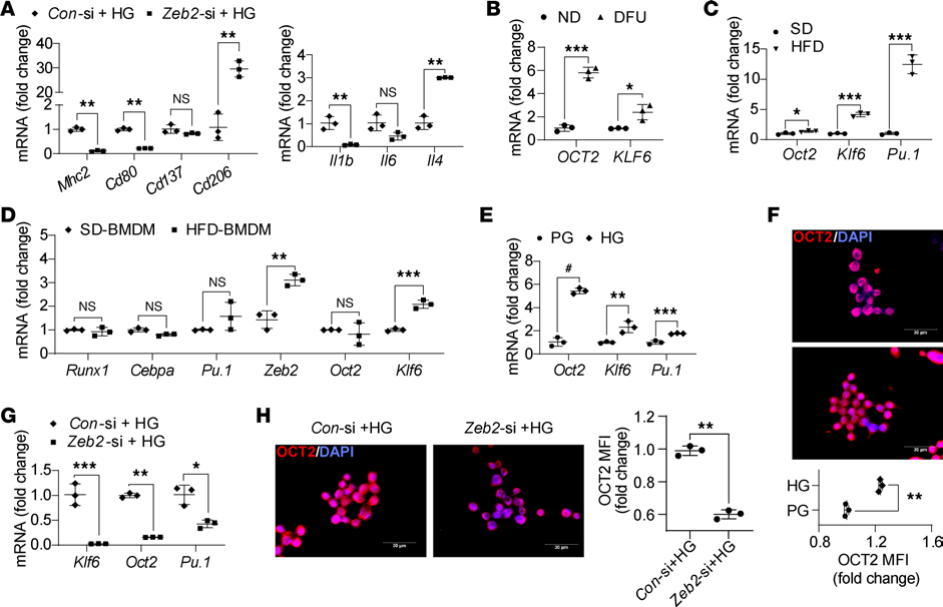
*Zeb2* silencing diminishes polarity of murine macrophages. Downregulation of proinflammatory status measured by (**A**) relative mRNA expression profile in *Zeb2*-silenced (100 nM *Zeb2* siRNA) HG-treated RAW264.7 cells. **P < 0.01 by paired, 2-tailed Student’s *t* test. Relative mRNA expression of MLDTF markers in (**B**) human wound patients’ samples, (**C**) d7 mouse wound tissue, and (**D**) BMDMs isolated from SD- and HFD-fed mice were assessed by qPCR. *P < 0.05, **P < 0.01, ***P < 0.001 by paired, 2-tailed Student’s *t* test. (**E**) Relative MLDTF mRNA expression and (**F**) immunostaining of OCT2 protein (scale bars: 30 μm, *n* = 3) also assessed in HG-treated RAW264.7 cells. **P < 0.01, ***P < 0.001, ^#^*P* < 0.0001 by paired, 2-tailed Student’s *t* test. MLDTF expression was determined by (**G**) relative mRNA expression analysis and (**H**) immunostaining images for OCT2 (scale bars: 30 μm, *n* = 3) in *Zeb2*-inhibited HG-treated macrophages. **P < 0.01, ***P < 0.001, ^#^*P* < 0.0001 by paired, 2-tailed Student’s *t* test. Experiments were repeated 3 times independently. Data are expressed as mean ± standard deviation. NS, not significant; ND, nondiabetic; DFU, diabetic foot ulcer; SD, standard diet; HFD, high-fat diet; PG, physiological glucose level; HG, hyperglycemia.

To comprehend the impact of hyperglycemia on ZEB2 expression and the inflammatory state in macrophages, murine RAW264.7 macrophages were treated with high glucose (HG, 25 mM) and compared with cells maintained at a physiological glucose (PG) level (5 mM). We observed a surge in ZEB2 protein expression accompanied by NF-κB activation (Figure 1, E and F). In addition, the gene expression profiles of various inflammatory factors (Supplemental Figure 1, E and F) showed elevated levels of proinflammatory cytokines monocyte chemoattractant protein-1 (MCP1), induced nitrogen oxide synthase (iNOS), and IL-1β (Figure 1G and Supplemental Figure 1G) compared with antiinflammatory cytokines such as Arginase 1 (ARG1) and IL-10 (Supplemental Figure 1H), indicating a proinflammatory phenotype in hyperglycemic macrophages. To further investigate macrophage polarization in response to HG, we conducted flow cytometric analysis of M1 (CD80^+^, CD86^+^, iNOS^+^) and M2 (CD163^+^) phenotypic markers. A substantial increase in the CD80^+^CD163^–^ (56.46 ± 2.61-fold), CD86^+^ (13.83 ± 0.46-fold), and iNOS^+^ (73.5 ± 1.96-fold) macrophage populations was observed in HG-treated RAW264.7 cells compared with the control (Figure 1, H and I). Furthermore, HG treatment significantly increased the gene (Supplemental Figure 1I) and protein (Figure 1J and Supplemental Figure 1J) levels of proinflammatory cytokines even in IL-4–induced macrophages. Interestingly, the HG-induced proinflammatory response was abolished in *Zeb2*-silenced macrophages, as demonstrated by the decrease in proinflammatory marker (*Mhc2*, *Cd80*, *Cd137*, *Il1b*, and *Il6*) and increase in antiinflammatory marker (*Cd206*, *Il4*) expression (Figure 2A).

The expression of lineage-specifying and lineage-determining genes in hematopoietic stem cells (HSCs) varies dynamically in response to microenvironmental cues, guiding the generation of diverse cellular phenotypes (13). To examine the impact of hyperglycemia on HSC lineage differentiation, we looked at the expression patterns of major myeloid lineage–directed transcription factors (MLDTFs), such as *Pu.1*, *Klf6*, *Oct2*, *Runx1*, and *Cebpa*. Human diabetic wound tissues exhibited a marked increase in *OCT2* and *KLF6* expression (Figure 2B), which was consistent with the elevated expression of *Oct2*, *Klf6*, and *Pu.1* in peripheral wounds of diabetic mice (Figure 2C), but no significant changes were detected in *Runx1* and *Cebpa* expression (Supplemental Figure 2A), suggesting enhanced myeloid lineage differentiation and polarization toward a proinflammatory wound environment. To understand the effect of hyperglycemia on central hematopoietic sources, we examined MLDTF and *Zeb2* expression in differentiated bone marrow–derived macrophages (BMDMs) from SD- and HFD-fed mice. *Zeb2* and *Klf6* expression was significantly upregulated under diabetic conditions, while other factors were unaffected (Figure 2D), suggesting a potential role in diabetes-associated transcriptional reprogramming. Correspondingly, elevated glucose levels significantly upregulated *Oct2*, *Klf6*, and *Pu.1* gene expression (Figure 2E) and OCT2 protein expression (Figure 2F), while the expression of other myeloid regulators, *Runx1* and *Cebpa*, remained unaltered (Supplemental Figure 2B). Surprisingly, *Zeb2* silencing notably diminished HG-induced MLDTF expression at both the gene and protein level (Figure 2, G and H). Collectively, these findings highlight a potential regulatory role for ZEB2 in modulating MLDTFs and warrant further mechanistic investigation.

### Zeb2 silencing shields macrophages from HG-induced inflammation by blocking proinflammatory phenotype.

**Figure 3:**
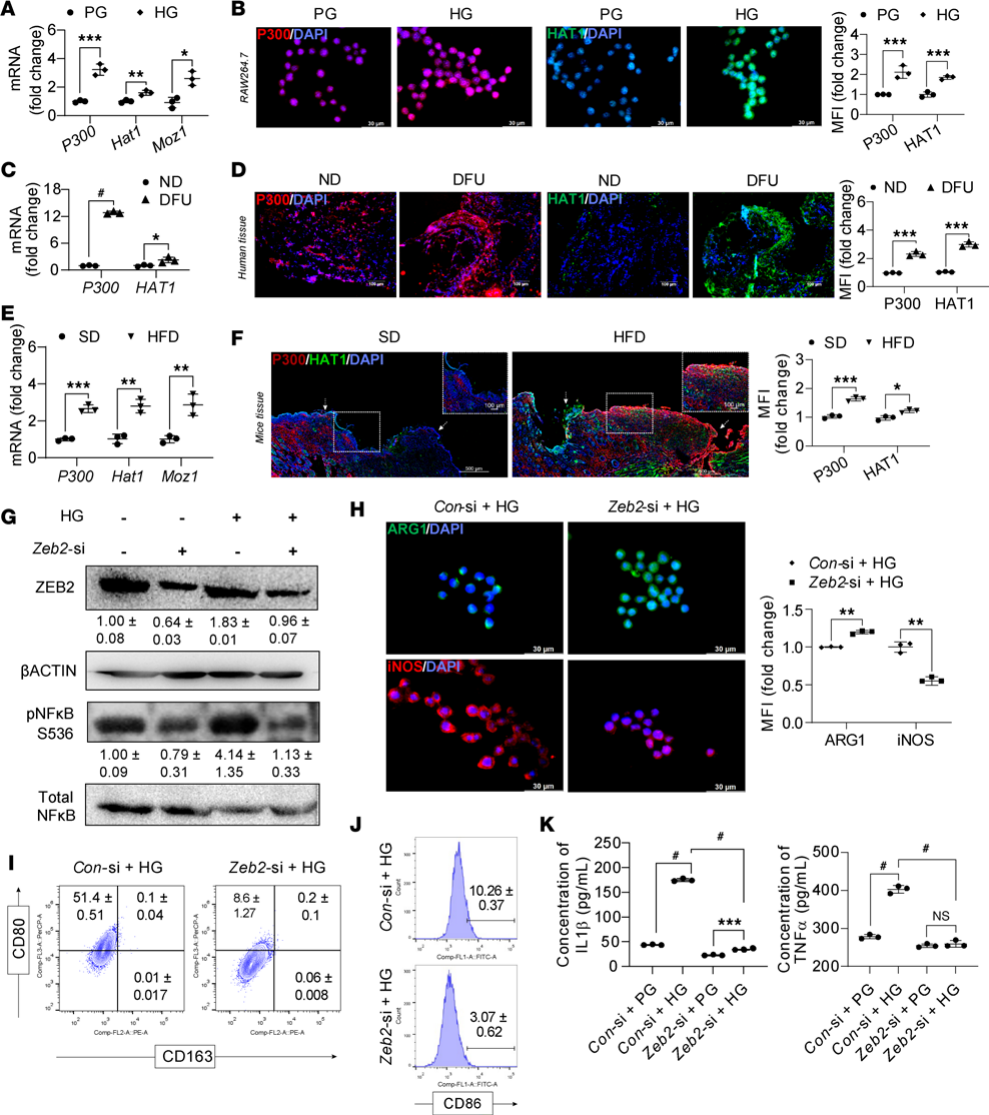
Inhibiting Zeb2 safeguards against hyperglycemia-induced macrophage inflammation and prevents their polarization. Expression profile of HATs upon induction with HG was determined based on (**A**) relative mRNA abundance by qPCR and (**B**) immunostaining for P300 and HAT1 (scale bars: 30 μm, *n* = 3). *P < 0.05, **P < 0.01, ***P < 0.001 by paired, 2-tailed Student’s *t* test. Expression profile of HATs in human wound patient samples by (**C**) mRNA expression and (**D**) tissue immunostaining for P300 and HAT1 (scale bars: 100 μm, *n* = 3/group). *P < 0.05, ***P < 0.001, ^#^*P* < 0.0001 by paired, 2-tailed Student’s *t* test. HAT expression in d7 mouse wound tissue by (**E**) relative mRNA expression and (**F**) tissue immunostaining with P300 (red) and HAT1 (green) antibodies (scale bars: 100 μm [higher magnification] and 500 μm [lower magnification]; *n* = 3/group). *P < 0.05, **P < 0.01, ***P < 0.001 by paired, 2-tailed Student’s *t* test. (**G**) The effect of *Zeb2* inhibition was assessed by Western blot analysis of ZEB2 and p-NF-κB. The reduction in proinflammatory status upon *Zeb2* inhibition revealed by (**H**) immunostaining (scale bars: 30 μm, *n* = 3) and (**I** and **J**) flow cytometric analysis of macrophage surface markers (*n* = 3). **P < 0.01 by paired, 2-tailed Student’s *t* test. (**K**) Culture media were analyzed for IL-1β and TNF-α by ELISA in control siRNA (*Con*-si) + HG and *Zeb2* siRNA (*Zeb2*-si) + HG groups (*n* = 3). ***P < 0.001, ^#^*P* < 0.0001 assessed with 1-way ANOVA followed by Tukey’s post hoc test. Experiments were repeated 3 times independently. Data are expressed as mean ± standard deviation. NS, not significant; ND, nondiabetic; DFU, diabetic foot ulcer; SD, standard diet; HFD, high-fat diet; PG, physiological glucose level; HG, hyperglycemia.

Chronic hyperglycemia perturbs glucose metabolism, resulting in elevated acetyl-CoA levels (14), and promotes proinflammatory gene expression primarily through HATs such as P300 (15) and HAT1 (16). The HATs P300, HAT1, and monocytic zinc finger 1 (MOZ1) play a crucial role in promoting NF-κB–mediated inflammatory responses (17–19). Therefore, we sought to analyze the expression profiles of different HATs under hyperglycemic conditions and investigate how they might influence the expression of ZEB2 in the wound microenvironment. A significant upregulation of *P300*, *Hat1*, and *Moz1* gene expression, as well as P300 and HAT1 protein expression, was found in macrophages exposed to HG conditions (Figure 3, A and B). Similar elevations were observed in the wound tissue from patients with DFU (Figure 3, C and D) and in diabetic mice (Figure 3, E and F), relative to their respective controls. Differentiated BMDMs derived from HFD mice also showed an upward trend in *P300* and *Moz1* RNA expression (Supplemental Figure 3A). Previous quantitative acetylome analysis revealed substantial hyperacetylation of multiple proteins, including the p65 subunit of NF-κB at the Lys310 residue, under HG conditions (20). This modification was strongly associated with the proinflammatory phenotype observed in the macrophage population (21).

To investigate the impact of ZEB2 on macrophage lineage and its inflammatory state, cells were transfected with either control siRNA or *Zeb2* siRNA (Supplemental Figure 3B). Silencing *Zeb2* expression led to a reduction in MLDTF mRNA (*Oct2*, *Klf6*, and *Pu.1*) and OCT2 protein levels (Figure 2G and H), and was associated with decreased activation of NF-κB (Figure 3G and Supplemental Figure 3C), which reconfirms that NF-κB directly regulates PU.1 and OCT2 (22, 23). Furthermore, *Zeb2*-silenced cells exhibited a marked decrease in proinflammatory markers alongside an increase in antiinflammatory markers, observed at both the gene and protein level (Figure 2A, Figure 3H, and Supplemental Figure 3D). Flow cytometric analysis of CD80^+^CD163^–^ (Figure 3I and Supplemental Figure 3E), CD86^+^, and iNOS^+^ (Figure 3J and Supplemental Figure 3F) macrophage populations, along with ELISA measurements of TNF-α and IL-1β levels (Figure 3K), further confirmed the reduction in proinflammatory markers following *Zeb2* silencing in hyperglycemic conditions (Figure 3K). Building on previous research that showed elevated ZEB2 expression reduced the NF-κB–mediated increase in proinflammatory cytokines triggered by LPS (24), whereas chronic TNF-α stimulation led to *Zeb2* upregulation (25), our study uncovers what we believe is a new insight: silencing *Zeb2* expression withdraws the hyperglycemia-induced proinflammatory state in macrophages.

### Pathogenic wound complications alleviated in Zeb2-ablated diabetic mice.

**Figure 4:**
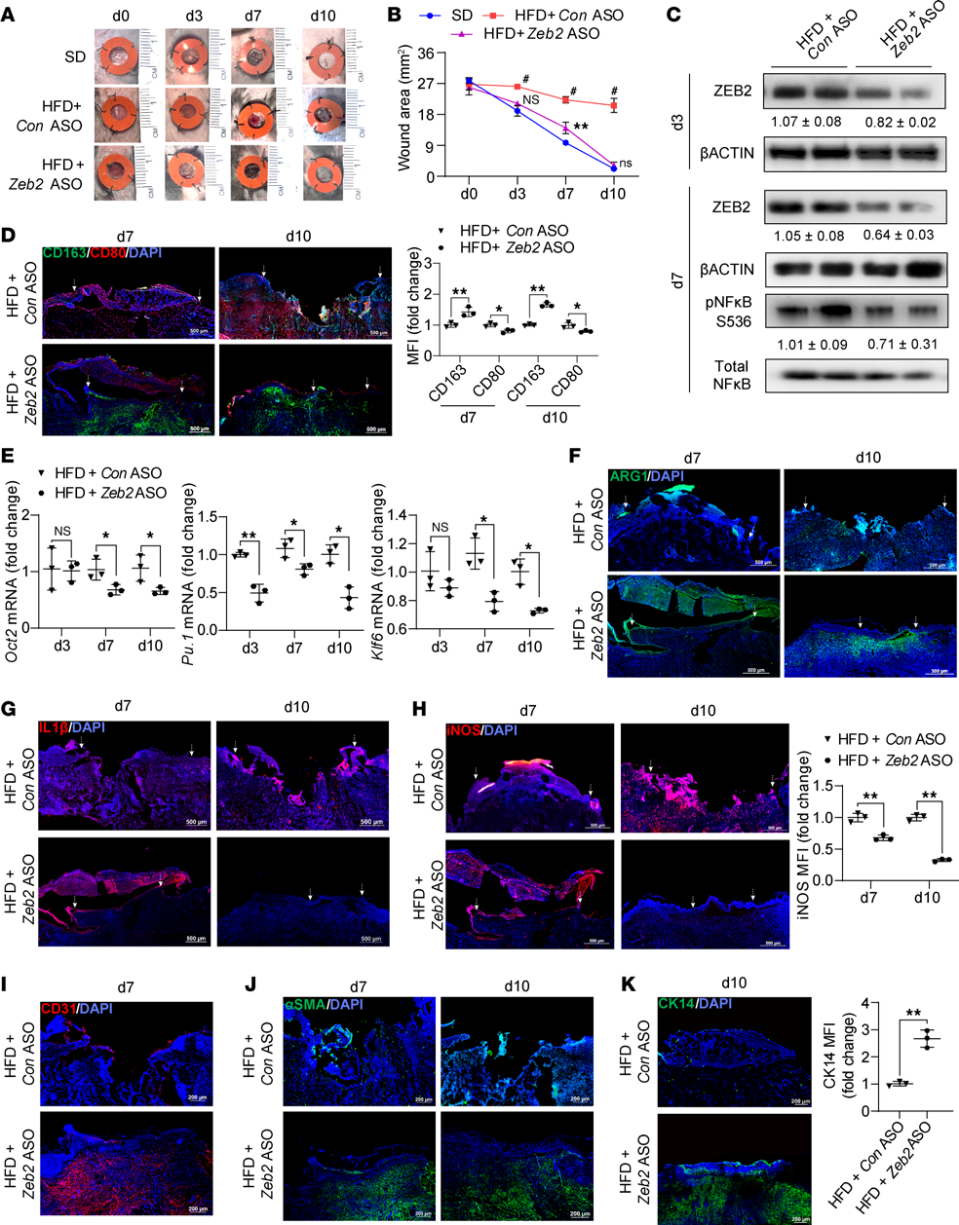
The suppression of Zeb2 ameliorated wound-healing complications in STZ-induced HFD mouse model. (**A**) Gross photographs of wounds (*n* = 3 mice/group) during healing at different time points in different groups. (**B**) Healing curve showing wound size of original wound versus time in days (*n* = 3 mice/group). **P < 0.01, ^#^*P* < 0.0001 by paired, 2-tailed Student’s *t* test. (**C**) Expression of ZEB2 and p-NF-κB protein assessed by Western blot in d3 and d7 mouse wound tissue. (**D**) Inflammatory status of the wound tissue assessed by the immunostaining for CD163 (green) and CD80 (red) in diabetic groups treated with *Con* ASO and *Zeb2* ASO (250 μM/100 μL/wound) (scale bars: 500 μm, *n* = 3 mice/group). *P < 0.05, **P < 0.01 by paired, 2-tailed Student’s *t* test. (**E**) Reduced MLDTF expression upon *Zeb2* inhibition in d10 murine wound tissue was assessed by relative mRNA expression. *P < 0.05, **P < 0.01 by paired, 2-tailed Student’s *t* test. Immunostaining for (**F**) ARG1, (**G**) IL-1β, and (**H**) iNOS was performed in d3 and d7 wound tissue to represent the inflammatory status upon *Zeb2* inhibition at the wound site (scale bars: 500 μm, *n* = 3 mice/group). **P < 0.01 by paired, 2-tailed Student’s *t* test. Representative immunostaining images for (**I**) CD31 in d7, (**J**) αSMA in d7 and d10, and (**K**) CK14 in d10 mouse wound tissue for neoangiogenesis, remodeling, and reepithelialization status in the wound tissue upon *Zeb2* inhibition (scale bars: 200 μm, *n* = 3 mice/group). **P < 0.01 by paired, 2-tailed Student’s *t* test. Data are expressed as mean ± standard deviation. NS, not significant; SD, standard diet; HFD, high-fat diet.

The effect of *Zeb2* inhibition was assessed in a wound model using STZ-administered HFD mice. Intradermal administration of *Zeb2* antisense oligonucleotides (ASOs) at the wound edge led to a significant reduction in *Zeb2* expression (Supplemental Figure 4A). Suppression of *Zeb2* expression in these mice was associated with improved healing (Figure 4A), confirmed by a reduction in wound bed area (Figure 4B) observed through H&E staining (Supplemental Figure 4B) compared with diabetic mice. *Zeb2* inhibition displayed a substantially intact epidermis and dermis, with an organized fibrous collagen structure, resembled the histological features of normal skin. The presence of developing hair follicles indicated successful healing of the dermal layer. Downregulation of ZEB2 protein expression (Figure 4C and Supplemental Figure 4C) coincided with reduced NF-κB activation (Figure 4C) in post-wound tissue (d3 and d7) of *Zeb2* ASO–treated diabetic mice, promoting inflammation resolution and enhancing wound healing. Administration of *Zeb2* ASO at the wound edge notably reduced proinflammatory macrophage accumulation by d10 after wounding compared with diabetic controls (Figure 4D). These findings indicate that, although diabetic wounds typically exhibit persistent inflammation, *Zeb2* suppression effectively attenuates the inflammatory response, reducing the proinflammatory burden during healing, as evidenced by the decreased levels of *Cd80* and *Cd86* (M1 macrophage markers) and increased levels of *Cd206* and *Cd163* (M2 macrophage markers) (Supplemental Figure 4D). On d7 after wounding, *Zeb2* ASO treatment significantly downregulated the gene expression profile of MLDTFs (*Oct2*, *Pu.1*, and *Klf6*) (Figure 4E) and proinflammatory cytokines (*Il1b*, *iNos*, *Il6*, and *Tnfa*), while markedly upregulating antiinflammatory cytokines (*Il4* and *Il10*) (Supplemental Figure 4E) compared with diabetic controls. Immunohistochemical analysis further demonstrated a significant elevation of ARG1 (Figure 4F and Supplemental Figure 4F), and time-dependent suppression of IL-1β (Figure 4G and Supplemental Figure 4G) and iNOS expression within the wound microenvironment (Figure 4H). This shift in macrophage phenotype indicates a move toward a more reparative and less inflammatory environment, which contributed to the improved healing process in diabetic wounds.

In wounds, the M2 macrophage phenotype is known to foster an environment, promotes new blood vessel formation, aiding tissue regeneration and accelerating tissue repair (26). In this context, examining the effect of *Zeb2* inhibition on proangiogenic markers could provide valuable insights into chronic diabetic wound healing. Suppression of *Zeb2* expression led to a marked increase in mRNA expression of key angiogenic factors *Tgfb*, *Cd31*, and *Cdh5* (VE-cadherin) on d7 and d10 after wounding (Supplemental Figure 4H), suggesting a correlation between ZEB2 levels and neovascularization. Immunohistochemical staining of d7 post-wound tissue showed a considerable increase in CD31 levels in *Zeb2* ASO–treated diabetic mice compared with control ASO–treated (*Con* ASO–treated) diabetic mice (Figure 4I and Supplemental Figure 4I). Similarly, α-smooth muscle actin (αSMA) levels were notably elevated in wound tissues of *Zeb2* ASO–treated diabetic mice on d7 and d10 (Figure 4J and Supplemental Figure 4J). The substantial increase in cytokeratin 14 (CK14) levels in *Zeb2* ASO–treated diabetic mouse tissue on d10 suggests a robust and developed epithelial layer compared with *Con* ASO–treated diabetic mice, which exhibited a thinner, less mature epithelial tissue, indicating delayed wound healing (Figure 4K). *Zeb2* ASO treatment in diabetic wound models shows exceptional promise for accelerating wound closure and enhancing tissue regeneration, providing an advanced strategy for treating chronic nonhealing diabetic wounds.

### Delivery of an acetylation inhibitor abrogates hyperglycemia-induced Zeb2 mRNA expression and macrophage inflammation.

**Figure 5:**
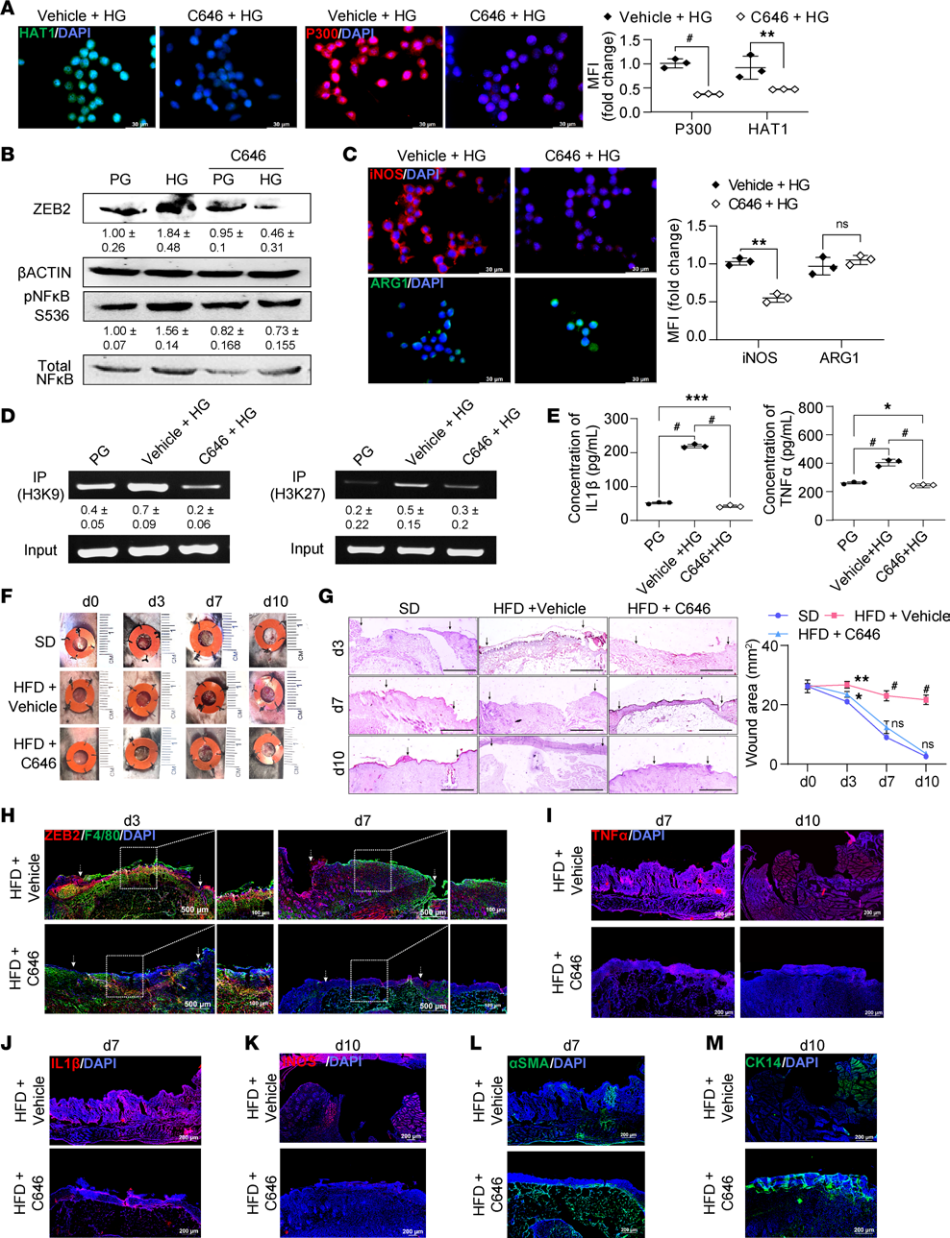
Inhibition of acetyltransferase activity ameliorated proinflammatory burden in hyperglycemic macrophages, even in diabetic wounds. Effect of C646 was assessed by (**A**) HAT1 and P300 by immunostaining (scale bars: 30 μm, *n* = 3) and (**B**) Western blot of ZEB2 and p-NF-κB (*n* = 3) in RAW264.7 cells upon C646 incubation (20 μM) in HG condition. **P < 0.01, ^#^*P* < 0.0001 by paired, 2-tailed Student’s *t* test. (**C**) Inflammatory status was measured by immunostaining of C646 + HG–treated RAW264.7 cells with iNOS and ARG1 antibodies (scale bars: 30 μm, *n* = 3). **P < 0.01 by paired, 2-tailed Student’s *t* test. (**D**) ChIP fold enrichment was assessed by ChIP-PCR for H3K9Ac and H3K27Ac activity at the ZEB2 promoter in PG-, vehicle + HG–, and C646 + HG–treated cells (*n* = 3). (**E**) Cytokine expression profile in culture media was analyzed for IL-1β and TNF-α by ELISA (*n* = 3). *P < 0.05, ***P < 0.001, ^#^*P* < 0.0001 by 1-way ANOVA followed by Tukey’s post hoc test. (**F**) Photographs of wound healing at different time points in SD, HFD + vehicle, and HFD + C646 groups (*n* = 3 mice/group). (**G**) H&E staining of wound images (scale bars: 1000 μm, *n* = 3 mice/group) and healing curve showing of original wound size versus time in days (*n* = 3 mice/group). **P < 0.01, **P < 0.01 by paired, 2-tailed Student’s *t* test. (**H**) Immunostaining of mouse wound tissue with ZEB2 (red) and F4/80 (green) antibodies in HFD group treated with or without C646 (10 μg/wound) (scale bars: 100 μm [higher magnification] and 500 μm [lower magnification]; *n* = 3 mice/group). *P < 0.05 by paired, 2-tailed Student’s *t* test. Inflammatory status was assessed by immunostaining for (**I**) TNF-α in d7 and d10, (**J**) IL-1β in d7, and (**K**) iNOS in d10 mouse wound tissue (scale bars: 200 μm, *n* = 3 mice/group). The remodeling phase was examined by immunostaining for (**L**) αSMA in d7 and (**M**) CK14 in d10 mouse wound tissue (scale bars: 200 μm, *n* = 3 mice/group). Data are expressed as mean ± standard deviation. NS, not significant; SD, standard diet; HFD, high-fat diet; PG, physiological glucose level; HG, hyperglycemia.

Histone acetylation is a key epigenetic modification that alters chromatin architecture through weak electrostatic interaction with DNA, leading to increased gene transcription. We found that under hyperglycemic conditions, macrophages in both murine and human wound beds showed higher expression of HATs (Figure 3, A–F), which coincided with altered expression of several genes, including *ZEB2* (27), contributing to wound chronicity. To have direct evidence for the role of specific HATs in hyperglycemia-induced *Zeb2* expression and inflammation in macrophages, we used C646, a potent and reversible competitive acetylation inhibitor with strong selectivity for P300 over other HATs (28). Treatment with C646 resulted in a significant reduction in the expression of P300 and HAT1 in macrophages exposed to hyperglycemia (Figure 5A). The C646 incubation markedly downregulated hyperglycemia-induced ZEB2 expression and NF-κB activation, as evidenced by immunoblot and immunocytochemical analyses (Figure 5B and Supplemental Figure 5A). In addition, immunocytochemical analysis revealed that C646 treatment markedly reduced iNOS and IL-1β levels in hyperglycemia-exposed macrophages, with no significant changes in ARG1 and MCP1 (Figure 5C and Supplemental Figure 5B). Chromatin immunoprecipitation (ChIP) assays demonstrated a pronounced increase in H3K9 hyperacetylation under hyperglycemic conditions; however, C646 treatment decreased the acetylation signature at the *ZEB2* promoter (Figure 5D), subsequently reducing its expression in the hyperglycemic state (Figure 5B). Furthermore, C646 treatment significantly reduced the gene expression profiles of proinflammatory markers and MLDTFs in macrophages (Supplemental Figure 5, C and D) and decreased the release of proinflammatory cytokines IL-1β and TNF-α under hyperglycemic conditions (Figure 5E). These findings indicate that C646-mediated suppression of histone acetylation in the *ZEB2* promoter prevented the hyperglycemia-induced proinflammatory state in macrophages.

Intradermal delivery of C646 to the wound edge in diabetic mice enhanced healing (Figure 5F) despite diabetes-associated impediments showing a marked reduction in wound size over time. Histological H&E staining of wound tissue cross sections showed a progressive reduction in wound bed depth in C646-treated diabetic mice compared with untreated diabetic controls (Figure 5G). By d7 after wounding, the C646-treated group displayed a thin layer of hyperplastic epidermis, whereas wounds in the untreated diabetic group remained fully open with no epidermal formation. Moreover, there was significant accumulation of inflammatory cells in the wounds of mice on d7, which was considerably reduced in C646-treated diabetic mice (Figure 5G). C646-treated diabetic wounds exhibited significantly lower ZEB2 expression compared with untreated diabetic wounds, as evidenced by immunohistochemical (Figure 5H and Supplemental Figure 6A) and Western blot analyses (Supplemental Figure 6B). Notably, the suppression of NF-κB activation in d10 post-wound tissues (Supplemental Figure 6C) provides mechanistic insight into the diminished cytokine production. The healing efficacy was evident through the downregulation of proinflammatory marker gene expression (Supplemental Figure 6D) and cytokine expression profile over the same time period (Supplemental Figure 6E). Immunohistochemical analysis revealed a downregulation of TNF-α on d7 and d10 (Figure 5I and Supplemental Figure 6F), IL-1β on d7 (Figure 5J and Supplemental Figure 6G), and iNOS on d10 (Figure 5K and Supplemental Figure 6H) post-wounding tissue following C646 administration. By d7 after wounding, the C646-treated diabetic mice demonstrated substantial healing progress with increased expression of αSMA and complete epidermal closure (Figure 5L and Supplemental Figure 6I). The reepithelialization of d10 wound tissue was evident by CK14 immunostaining, unlike the untreated diabetic groups (Figure 5M and Supplemental Figure 6J).

In summary, our findings highlight the critical insight of epigenetic regulation of *ZEB2* in regulating chronic diabetic wound inflammation. Thus, suppression of *Zeb2* utilizing ASOs or small-molecule HAT inhibitors in diabetic wounds potentially reduces the inflammatory burden at the wound site and expedites the healing process.

## Discussion

Macrophages play a central role during wound healing through a coordinated sequence of events, seamlessly adapting to the nuanced temporal and spatial cues (29). Numerous studies have reported the highly unregulated and persistent inflammation predominantly driven by activated macrophages (5, 30), dysregulated by hyperglycemia, hyperlipidemia, hypoxia, and other environmental stressors. Our study delves into the proinflammatory effects of HG on macrophages (31, 32), which are intricately modulated by ZEB2, a transcription factor widely expressed across hematopoietic lineages (33) essential for maintaining the tissue-specific macrophage identities (4). Previously studies have shown that elevated TNF-α levels induce ZEB2 expression, intensifying proinflammatory responses by binding to E-box elements within cytokine gene promoters (25). NF-κB–regulated MLDTFs like *Oct2*, *Klf6*, and *Pu.1* contribute to sustaining the proinflammatory state of macrophage lineages (23, 34–38). Chronic inflammation in hyperglycemic conditions has also been linked to enhanced lysine acetylation, mediated by HATs and non-HATs, resulting in aberrant gene expression (15). For instance, NF-κB activation in response to hyperglycemia promotes proinflammatory gene transcription via the P300/Creb-binding protein (CBP) chromatin-modifying complex (19, 39, 40). This effect was linked to P300/CBP acetyltransferase activity, which is known to cause SIRT6 acetylation, disrupting SIRT6-FOXA2 interactions with the *ZEB2* promoter and activating its transcription (27). Based on these findings, we hypothesized that reducing ZEB2 expression by inhibiting P300/CBP acetyltransferase activity could alleviate the inflammatory burden in hyperglycemic macrophages.

In our in vitro and in vivo animal studies, hyperglycemic wound macrophages showed elevated HAT activity, which were also increased in BMDMs from diabetic mice, indicating that hyperglycemia-driven acetylome programming potentially originates at the central hematopoietic level (31) and is subsequently propagated to peripheral tissues. The upregulation of *Zeb2* in differentiated BMDMs from HFD-fed mice further reflects diabetes-induced reprogramming that begins centrally and extends beyond the periphery. However, future in-depth study is needed to investigate hyperglycemia-induced epigenetic modifications, including histone acetylation of *Zeb2* in BM cells, which would provide a comprehensive mechanistic understating and link these molecular changes to impaired wound healing process. Increased acetyl-CoA availability during chronic hyperglycemic state, primarily due to enhanced fatty acid oxidation (10, 14), and the recruitment of transcription factors, including NF-κB at the *ZEB2* promoter, offer possible explanations linking hyperglycemia to the induction of acetylases causing putative acetylation at the specific loci; however, the precise regulatory code guiding acetylases to specific genomic loci under hyperglycemic conditions remains poorly understood and therefore highlights a key gap for future investigation.

We silenced *Zeb2* expression using siRNA and examined the exclusive role of the hyperglycemia-induced proinflammatory state of macrophages. A notable reduction in MLDTF expression was noticed in *Zeb2*-silenced macrophages associated with reduced NF-κB activation, clarifying the participation of ZEB2 in regulating inflammation under hyperglycemia-induced molecular chaos via NF-κB. Intradermal administration of *Zeb2* ASO in diabetic mice notably exacerbated diabetes-related wound healing complications, as indicated by the inhibition of prolonged proinflammatory status and induction of antiinflammatory markers in proliferative and remodeling stages of wound healing processes. Interestingly, we discovered that administering *Zeb2* ASO improved wound healing complications, evidenced by the appearance of granular tissue, full thickness epidermal layers, and propagation of fibroblast cells in the wound bed microenvironment. These findings suggest that inhibiting ZEB2 expression in the wound microenvironment holds promising therapeutic potential for managing diabetes-associated wound complications.

Recognizing the challenges associated with delivering ASOs to dermatologic wounds due to the skin’s barrier function and altered architecture of wounded tissue, and based on our observation that hyperglycemia-induced ZEB2 expression is mediated by the p300/CBP acetyltransferase, we propose C646 as an alternative therapeutic approach. We used C646 as a pharmacological intervention because of its widely reported selectivity toward P300/CBP (28), which serve as central coactivators that integrate diverse signaling inputs and act as hubs for chromatin acetylation, particularly at H3K9Ac and H3K27Ac. Delivery of small molecules is advantageous due to their ease of tissue penetration, stability, cost effectiveness, and rapid action. Intradermal C646 administration notably reduced proinflammatory burden in the wound of diabetic mice. Inhibition of P300/CBP activity was reflected in the reduction in NF-κB activity along with the proinflammatory markers and MLDTF expression in treated macrophages. In addition, ChIP-qPCR assays demonstrated that hyperglycemia provokes extensive histone core hyperacetylation, characterized by marked enrichment of H3K9 and H3K27 acetylation at the *ZEB2* promoter region (chr2: 45113049–45113250), thereby augmenting its transcriptional activity and propagating chronic inflammatory responses. However, silencing individual HATs or employing broad-spectrum HAT inhibitors could help delineate the relative contribution of each HAT to ZEB2-driven inflammatory responses in hyperglycemic macrophages.

Moreover, C646-treated wounds showed a progressive decrease in ZEB2 expression over time, accompanied by a reduction in MLDTF-driven macrophages, leading to the sustained inactivation of classical pathways involved in wound healing proliferation and remodeling (41, 42). In contrast, the inactivation of P300/CBP did not affect the expression profile of antiinflammatory markers. This observation indicates that treatment with the selective P300/CBP inhibitor C646 facilitates diabetic wound healing by alleviating hyperglycemia-induced inflammatory burden in macrophages. Nevertheless, future studies employing ATAC-seq or ChIP-seq could further uncover additional loci and genome-wide chromatin remodeling events associated with hyperglycemia.

In summary, our findings demonstrated that hyperglycemia-induced P300 HAT activity drives hyperactivation of ZEB2 expression in macrophages, exacerbating chronic inflammation in diabetic wounds. Treatment with either the P300/CBP inhibitor C646 or *Zeb2* ASO effectively alleviated wound complications in diabetic mice by curbing wound inflammation. Both C646- and *Zeb2* ASO–treated groups exhibited similar outcomes, suggesting that lowering the acetylation profile in the wound bed ameliorates ZEB2-driven chronic inflammation. Thus, our study uncovers a key role for ZEB2 in driving hyperglycemia-induced persistent inflammation in macrophages, positioning it as a potential therapeutic target for managing diabetic chronic wounds. However, further research on wound-macrophage-specific ZEB2-haplodeficient transgenic animal models will provide direct evidence of ZEB2’s role in wound macrophage biology and its implications for wound management.

## Methods

### Sex as a biological variable.

Our study on C57BL/6J mice included only males, as hormonal factors in females provide protection against oxidative stress and STZ-induced pancreatic β cell damage. For human studies, we examined both male and female patients, and similar findings are reported for both sexes.

### Mouse models and treatments.

WT C57BL/6J male mice, 4–5 weeks old, and weighing between 18 and 22 g, were sourced from the IISER Mohali animal facility. They were acclimated for 5–6 days at the NIPER Mohali animal house under a controlled environment, which included a 12-hour light/dark cycle, a temperature of 23°C ± 2°C, and relative humidity of 55% ± 5%. The mice were provided with a standard pellet diet and water ad libitum. For the development of type 2 diabetic mouse model, C57BL/6J mice were injected with STZ (S0130, Sigma-Aldrich) intraperitoneally following a published protocol (43). After completion of the treatment, mice were fed with HFD pellets (D12492, Research Diet Inc.) having 60% kcal of fat for 4 weeks for developing diabetes-induced complications. All remaining mice were maintained on SD pellets containing 10% kcal from fat for a duration of 8 weeks. Throughout the experiment, the animals had unrestricted access to sterilized water and food. Blood glucose levels were consistently monitored using an Accu-Chek glucometer (Roche). All mice were subjected to rostral excisional wound following a published protocol (44). One group of (STZ-induced HFD) diabetic mice received intradermal delivery of C646 (SML0002, Sigma-Aldrich) at the wound edge at various time points. A total of 10 μg C646/wound was injected by resuspending in PBS in 3 different sites of the wound edge. PBS as vehicle was injected (70 μL) similarly on the 3 different sites on of wound edge of the control diabetic group. To evaluate the *Zeb2* ASO efficacy, diabetic mouse wound model was developed (45). One group of diabetic mice received with intradermal delivery of *Con* ASO and another group with *Zeb2* ASO at 250 μM complexing with Invivofectamine 3.0 Reagent (IVF3001, Invitrogen) at a total volume of 100 μL/wound for 3 days.

At certain intervals, wound images were captured and the closure area measured through ImageJ (NIH) analysis. Mice were then sacrificed and wound bed tissues were collected and processed for further experimental studies. Immediately after collection, tissues were processed and embedded in tissue freezing medium (14020108926, Leica Biosystems) for cryosectioning.

### Human participants.

A total of 18 diabetic and 6 nondiabetic (male and female) patients participated in this study. The study population was categorized into 2 groups based on BMI and blood glucose level. Study participants having a fasting blood glucose level (mmol/L) of less than 5 were considered the nondiabetic group (*n* = 5), whereas patients with a fasting blood glucose level (mmol/L) of greater than 7 were considered the diabetic group (*n* = 17), as presented in Supplemental Table 1. In this study, surgically wounded debridement tissue samples were collected from the patients who were admitted to the Dayanand Medical College & Hospital, Ludhiana, Punjab, and underwent surgery.

### BM cell extraction.

As previously reported, BM cells were isolated from the surgically removed the femur and tibia of SD- and HFD-fed mice in a sterile environment, followed by flushing out and differentiating into BMDMs (46). After removing the epiphyses, the marrow cavities were carefully cleaned using complete media (DMEM [A5256801, Gibco] with 10% heat-inactivated FBS, 1% penicillin-streptomycin solution [15140122, Gibco]) and collected in a 50 mL tube. After centrifuging the collected BM cells at 200*g* for 5 minutes, the pellet was treated with cold RBC lysis buffer for 5 minutes, followed by another centrifugation at 200*g* for 5 minutes at 4°C, then resuspended in growth media (complete media containing 20 ng/mL mouse recombinant macrophage colony–stimulating factor (M-CSF) protein (315-02-10UG, PeproTech/Gibco) and passed through a 100 μm sterile cell strainer to remove debris and macroparticles. After that, cells were seeded in 6-well plate at 1 × 10^6^ cells per well. Additional M-CSF–containing growth media was added on the third day after the seeding and cultured for 4 days and harvested for further experiments.

### Cell culture and treatments.

RAW264.7 macrophages were obtained from the National Centre for Cell Science (NCCS), Pune, India, and cultured in DMEM supplemented with 10% FBS (10082147, Gibco) and 1% penicillin-streptomycin solution at 37°C in a humidified atmosphere with 5% CO_2_. Briefly, macrophages were at a density of 8 × 10^4^ cells in 35-mm culture plates until they reached 90% confluence. A solution of 25 mM D-glucose (G7021, Sigma-Aldrich) in PBS (10010023, Gibco) was used for in vitro HG treatment, while DMEM containing 5 mM D-glucose (31885023, Gibco) was used to represent PG conditions. We filtered the solutions using 0.4 μm filters before treating the cells. C646 at 20 μM was used for in vitro treatment (47).

### Oligonucleotide transfection.

For transfection of *Zeb2* siRNA and the corresponding control siRNA, Lipofectamine RNAiMAX transfection reagent (13778-075, Invitrogen) was employed in accordance with the manufacturer’s instructions. Briefly, RAW264.7 macrophages were seeded at a density of 0.1 × 10^6^ cells per well in a 12-well plate containing antibiotic-free complete growth medium and incubated for 24 hours before transfection. In each well, 100 nM *Zeb2* siRNA or control siRNA was separately mixed with Lipofectamine RNAiMAX reagent in Opti-MEM Reduced Serum Medium (31985070, Gibco) and incubated for 5 minutes. The transfection mixture was then added to the cells in complete growth medium and incubated for 48 hours. Afterward, the cells were washed, fresh complete growth medium was added, and the cells were used for subsequent treatments (48).

### Immunocytochemistry.

Cells cultured on sterile glass coverslips overnight were subjected to treatment with or without C646 (20 μM), *Con* siRNA (100 nM), and *Zeb2* siRNA (100 nM) for 24 hours. After incubation, cells were rinsed with PBS and fixed using ice-cold methanol (65524, SRL), and permeabilized with 0.25% Triton X-100 (T8787, Sigma-Aldrich) in PBS at room temperature. Cells were then blocked with 1% BSA (MB083, Himedia) in PBS containing 0.1% Tween 20 (P1379, Sigma-Aldrich) for 30 minutes at room temperature and then incubated with primary antibodies for 1 hour at room temperature. After 3 washes with ice-cold PBST (E-IR-R310, Elabscience), the cells were exposed to fluorescence-conjugated secondary antibodies for 1 hour in the dark at room temperature. Prior to mounting, cells were washed 3 additional times with ice-cold PBST (5 minutes each) and then mounted onto glass slides using anti-fade mounting medium with DAPI (H-1500, Vector Laboratories) (48). Cellular images were captured using an inverted fluorescence microscope (Leica DMi8), and subsequent image analysis was performed using LAS X software. A comprehensive list of all antibodies, along with their catalog numbers and the dilutions employed in the experiments, is presented in Supplemental Table 2.

### Immunofluorescence.

After fixing in 10% neutral buffered formalin (HT501128, Sigma-Aldrich) overnight at 4°C, the wound tissue samples were processed and embedded in tissue freezing medium (14020108926, Leica Biosystems) at –80°C. Cryosectioning was carried out using a CM 1860 cryostat (Leica Biosystems). For immunostaining, 5-μm tissue cryosections were placed on gelatin-coated glass slides, fixed in ice-cold methanol, and then blocked with 5% BSA in a blocking buffer. The sections were incubated with specific primary antibodies for 1 hour at room temperature. Following washing with PBST, the samples were exposed to fluorescence-conjugated secondary antibodies and counterstained with an antifade mounting medium containing DAPI (48). Images were captured using a confocal microscope (Zeiss LSM 880) and analyzed with Zeiss ZEN lite software.

### H&E staining and imaging.

The tissue cryosections mounted on gelatin-coated glass slides were subjected to regressive staining (48). After proceeding with different percentages of alcohol, slides were subsequently rinsed with distilled water (dH_2_O), and then incubated in Harris Hematoxylin (HHS16, Sigma-Aldrich) for 3 minutes, followed by a 2-minute rinse in dH_2_O. The slides were further subjected to alcohol solution containing 0.3% acetic acid, rinsed in dH_2_O, and then dipped in 0.3% ammonium water to induce bluing, followed by another rinse in dH_2_O. The procedure continued with passages through 80% ethanol followed by staining with 2% Eosin (E4009, Sigma-Aldrich) for 30 seconds. Excess stain was removed by washing the sections in 95% ethanol for 20 seconds, and then in 100% ethanol for 20 seconds. Finally, the slides were briefly immersed in xylene before being mounting with DPX mounting medium (06522, Sigma-Aldrich). The resulting H&E-stained slides were examined using a Leica DMi8 microscope for imaging.

### RNA extraction and quantitative PCR.

Total RNA was isolated from cells using TRIzol Reagent (15596026, Invitrogen) (48). For tissue samples, 5 mg of murine and human wound tissue were homogenized with TRIzol using a TissueLyser II (Qiagen), followed by quantification with a NanoDrop OneC (Thermo Fisher Scientific). The extracted RNA (300 ng) was digested with DNase I (M0303S, New England Biolabs) and subsequently reverse transcribed with the iScript cDNA Synthesis Kit (1708891, Bio-Rad). Real-time quantitative PCR was carried out using PowerUp SYBR Green Master Mix (A25742, Applied Biosystems) on a QuantStudio 5 Real-Time PCR System (Applied Biosystems), employing gene-specific primers. Relative expression or fold change was calculated by the 2^ΔΔCt^ formula, normalizing to 18S rRNA. Different gene-specific primers, siRNAs, and ASOs are presented in Supplemental Table 3.

### Immunoblotting.

Cultured cells were lysed in NP40 cell lysis buffer (FNN0021, Invitrogen) augmented with Halt protease and phosphatase inhibitor cocktail (78440, Thermo Fisher Scientific) and subsequently centrifuged at 16,200*g* for 10 minutes at 4°C. Tissue samples from mouse and human wounds were homogenized using a TissueLyser II (Qiagen) in NP40 lysis buffer supplemented with the same inhibitor cocktail. Protein concentrations in the cell lysates were quantified using the Pierce BCA Protein Assay Kit (23225, Thermo Fisher Scientific), and 50 μg of protein from each sample was resolved in a 10% SDS-PAGE gel and transferred to Immun-Blot PVDF membranes (1620177, Bio-Rad) using a Turbo Blotting System (Bio-Rad). Membranes were blocked with 5% BSA in Tris-buffered saline containing 0.1% Tween 20 (TBST) for 1 hour, followed by primary antibody incubation overnight at 4°C. After 3 washes with TBST for 10 minutes each, membranes were probed with peroxidase-conjugated secondary antibodies for 2 hours at room temperature. Following an additional set of three 10-minute washes with TBST, the membranes were treated with Clarity Western ECL Substrate (1705061, Bio-Rad) and visualized using a Chemidoc XRS+ System (Bio-Rad) and analyzed with Image Lab software (48).

### ELISA.

Levels of IL-1β and TNF-α in the cell culture supernatants from both control and treated cells were assessed using mouse-specific ELISA kits for IL-1β (432604, BioLegend) and TNF-α (430907, BioLegend), in accordance with the manufacturer’s instructions.

### ChIP-PCR.

ChIP assay was performed using the SimpleChIP plus Enzymatic Chromatin IP kit, Magnetic Beads (9005, Cell Signaling Technology). Briefly, upon reaching confluence in 100-mm tissue culture plates, RAW264.7 cells were treated under PG, HG, and C646 + HG conditions. Following treatment, cells were processed according to the manufacturer’s protocol. Cross-linked and fragmented chromatin was immunoprecipitated using anti-H3K9Ac and anti-H3k27Ac antibodies. The purified DNA was subsequently analyzed by PCR using specific primers flanking the promoter region of *ZEB2*, and the PCR products were visualized by agarose gel electrophoresis. Band intensities were quantified using ImageJ software and the amount of immunoprecipitated DNA in each sample was represented as relative to the corresponding input chromatin.

### Flow cytometry.

After various treatments, cells were collected and washed with ice-cold PBS, followed by centrifugation at 0.2*g* for 5 minutes. The resulting cell pellet was incubated with TruStainFcX (anti-mouse CD16/CD32) antibody for 30 minutes at 4°C, then washed once with ice-cold PBS. Subsequently, the cells were incubated with antibodies against CD80, CD86, CD163, and iNOS for 1 hour. Following this incubation, the cells were washed with PBS and analyzed using the BD Accuri C6 Plus.

### Statistics.

Data analysis was carried out utilizing Prism software version 8.0 (GraphPad Software, Inc.). Results are expressed as mean ± standard deviation. Statistical significance was determined through paired, 2-tailed Student’s *t* test for 2 independent groups or 1-way ANOVA with Tukey’s post hoc test for multiple groups, with a *P* value of less than 0.05 considered indicative of significance. Each experiment in vitro was repeated 3 times independently.

### Study approval.

All animal experiments were performed following the guidelines prescribed by and with the approval of the Institutional Animal Ethics Committee (IAEC) of National Institute of Pharmaceutical Education and Research, S.A.S. Nagar, Punjab, India (protocol no. IAEC/22/37-M). All human participants provided written informed consent using documents reviewed and approved by Institute Ethics Committee (IEC) of Dayanand Medical College & Hospital, Ludhiana, Punjab, India (protocol no. DMCH/R&D/2023/172; IEC no. 2023-831).

### Data availability.

All data values underlying the figures and supplemental data are reported in the Supporting Data Values file.

## Author contributions

SR, D Patra, PR, SS, and D Pal were involved in investigation and validation. Formal analysis was conducted by SR, D Patra, and D Pal. SR, D Patra, SD, and D Pal contributed to manuscript review and editing. Study conceptualization and methodology were carried out by SR, D Patra, and D Pal. SR and D Pal drafted the original manuscript. AB, SD, KT, and D Pal provided resources. ChIP experiments were performed and reviewed by PK, KS, CKS, and D Pal. Funding acquisition and supervision were led by D Pal. All authors participated in manuscript review and editing. D Pal serves as the guarantor of this work, with full access to all study data and responsibility for the integrity and accuracy of the data analysis.

## Funding support

This work is the result of NIH funding, in whole or in part, and is subject to the NIH Public Access Policy. Through acceptance of this federal funding, the NIH has been given a right to make the work publicly available in PubMed Central.

- Department of Biotechnology (DBT)/Wellcome Trust India Alliance, India, grant no. IA/I/23/2/506994 (to D Pal).
- DBT, government of India, grant no. BT/PR40669/MED/32/761/2020 (to D Pal).
- NIH grant DK136814 (to KS).

## Supplementary Material

Supplemental data

Unedited blot and gel images

Supporting data values

## Version 1 01/29/2026

In-Press Preview

## Version 2 03/09/2026

Electronic publication

## References

[1] Akkus G, Sert M (2022). Diabetic foot ulcers: a devastating complication of diabetes mellitus continues non-stop in spite of new medical treatment modalities. World J Diabetes.

[2] Chen L (2018). Inflammatory responses and inflammation-associated diseases in organs. Oncotarget.

[3] Krzyszczyk P (2018). The role of macrophages in acute and chronic wound healing and interventions to promote pro-wound healing phenotypes. Front Physiol.

[4] Scott CL (2018). The transcription factor ZEB2 is required to maintain the tissue-specific identities of macrophages. Immunity.

[5] Scott CL, Omilusik KD (2019). ZEBs: novel players in immune cell development and function. Trends Immunol.

[6] Jiang N (2023). Acetylation in pathogenesis: revealing emerging mechanisms and therapeutic prospects. Biomed Pharmacother.

[7] Haery L (2015). Histone acetyltransferases and histone deacetylases in B- and T-cell development, physiology and malignancy. Genes Cancer.

[8] Ashburner BP (2001). The p65 (RelA) subunit of NF-kappaB interacts with the histone deacetylase (HDAC) corepressors HDAC1 and HDAC2 to negatively regulate gene expression. Mol Cell Biol.

[9] Uribarri J (2011). Restriction of advanced glycation end products improves insulin resistance in human type 2 diabetes: potential role of AGER1 and SIRT1. Diabetes Care.

[10] Giri B (2018). Chronic hyperglycemia mediated physiological alteration and metabolic distortion leads to organ dysfunction, infection, cancer progression and other pathophysiological consequences: An update on glucose toxicity. Biomed Pharmacother.

[11] Guo S, Dipietro LA (2010). Factors affecting wound healing. J Dent Res.

[12] Sharifiaghdam M (2022). Macrophages as a therapeutic target to promote diabetic wound healing. Mol Ther.

[13] Meng Y Nerlov C (2025). Epigenetic regulation of hematopoietic stem cell fate. Trends Cell Biol.

[14] Luo X (2016). Hyperglycemic stress and carbon stress in diabetic glucotoxicity. Aging Dis.

[15] Miao F (2004). In vivo chromatin remodeling events leading to inflammatory gene transcription under diabetic conditions. J Biol Chem.

[16] Gruber JJ (2019). HAT1 coordinates histone production and acetylation via H4 promoter binding. Mol Cell.

[17] Poziello A (2021). Recent insights into *Histone Acetyltransferase-1*: biological function and involvement in pathogenesis. Epigenetics.

[18] Chan EM (2007). MOZ and MOZ-CBP cooperate with NF-kappaB to activate transcription from NF-kappaB-dependent promoters. Exp Hematol.

[19] Mukherjee SP (2013). Analysis of the RelA:CBP/p300 interaction reveals its involvement in NF-κB-driven transcription. PLoS Biol.

[20] Zhang Y (2019). The pivotal role of protein acetylation in linking glucose and fatty acid metabolism to β-cell function. Cell Death Dis.

[21] Surace AEA, Hedrich CM (2019). The role of epigenetics in autoimmune/inflammatory disease. Front Immunol.

[22] Bonadies N (2010). PU.1 is regulated by NF-kappaB through a novel binding site in a 17 kb upstream enhancer element. Oncogene.

[23] Tamassia N (2021). Induction of OCT2 contributes to regulate the gene expression program in human neutrophils activated via TLR8. Cell Rep.

[24] Ding Q (2018). ZEB2 attenuates LPS-induced inflammation by the NF-κB pathway in HK-2 cells. Inflammation.

[25] Chua HL (2007). NF-kappaB represses E-cadherin expression and enhances epithelial to mesenchymal transition of mammary epithelial cells: potential involvement of ZEB-1 and ZEB-2. Oncogene.

[26] Rőszer T (2015). Understanding the mysterious M2 macrophage through activation markers and effector mechanisms. Mediators Inflamm.

[27] Zhao K (2023). MOF-mediated acetylation of SIRT6 disrupts SIRT6-FOXA2 interaction and represses SIRT6 tumor-suppressive function by upregulating ZEB2 in NSCLC. Cell Rep.

[28] Bowers EM (2010). Virtual ligand screening of the p300/CBP histone acetyltransferase: identification of a selective small molecule inhibitor. Chem Biol.

[29] Martinez FO, Gordon S (2014). The M1 and M2 paradigm of macrophage activation: time for reassessment. F1000Prime Rep.

[30] Lin S (2023). Wounds under diabetic milieu: the role of immune cellar components and signaling pathways. Biomed Pharmacother.

[31] Edgar L (2021). Hyperglycemia induces trained immunity in macrophages and their precursors and promotes atherosclerosis. Circulation.

[32] Ayala TS (2019). High glucose environments interfere with bone marrow-derived macrophage inflammatory mediator release, the TLR4 pathway and glucose metabolism. Sci Rep.

[33] Wu X (2016). Transcription factor Zeb2 regulates commitment to plasmacytoid dendritic cell and monocyte fate. Proc Natl Acad Sci U S A.

[34] Lu SC (2009). The essential role of Oct-2 in LPS-induced expression of iNOS in RAW 264.7 macrophages and its regulation by trichostatin A. Am J Physiol Cell Physiol.

[35] Dunn T (1996). Transcription factor Oct-2 is expressed in primary murine macrophages. Blood.

[36] Date D (2014). Kruppel-like transcription factor 6 regulates inflammatory macrophage polarization. J Biol Chem.

[37] Joo M (2008). Genetic determination of the role of PU.1 in macrophage gene expression. Biochem Biophys Res Commun.

[38] Li G (2020). Transcription factor PU.1 and immune cell differentiation (Review). Int J Mol Med.

[39] Bhatt D, Ghosh S (2014). Regulation of the NF-κB-mediated transcription of inflammatory genes. Front Immunol.

[40] Berghe WV (1999). The nuclear factor-kappaB engages CBP/p300 and histone acetyltransferase activity for transcriptional activation of the interleukin-6 gene promoter. J Biol Chem.

[41] Caputa G (2019). Macrophage metabolism: a wound-healing perspective. Immunol Cell Biol.

[42] Gordon S, Martinez FO (2010). Alternative activation of macrophages: mechanism and functions. Immunity.

[43] Furman BL (2021). Streptozotocin-induced diabetic models in mice and rats. Curr Protoc.

[44] Fischer KS (2023). Protocol for the splinted, human-like excisional wound model in mice. Bio Protoc.

[45] Rowland MB (2023). Assessing wound closure in mice using skin-punch biopsy. STAR Protoc.

[46] Patra D (2024). Adipose tissue macrophage derived microRNA-210-3p disrupts systemic insulin sensitivity by silencing GLUT4 in obesity. J Biol Chem.

[47] Roy S (2023). Oxidized pullulan exhibits potent antibacterial activity against S. aureus by disrupting its membrane integrity. Int J Biol Macromol.

[48] Patra D (2022). miR-210-3p promotes obesity-induced adipose tissue inflammation and insulin resistance by targeting SOCS1-mediated NF-κB pathway. Diabetes.

