# Supplementary material for: Hyperglycemia-induced P300/CBP acetyltransferase drives ZEB2-mediated proinflammatory macrophages and delays wound healing
Source: JCI Insight. 2026 Jan 29;11(5):e192146. doi: 10.1172/jci.insight.192146 (PMC13041667; doi:10.1172/jci.insight.192146)
Supplement: Supplemental data [file jciinsight-11-192146-s180.pdf]

Hyperglycemia-induced P300/CBP acetyltransferase drives ZEB2-mediated pro-inflammatory macrophages and delays wound healing

Soumyajit Roy[1], Debarun Patra[1,2], Palla Ramprasad[1], Shivam Sharma[3], Parul Katiyar[4], Ashvind Bawa[5], Kanhaiya Singh[4], Kulbhushan Tikoo[3], Suman Dasgupta[6], Chandan K Sen[4], Durba Pal[1,4*]

[1] Department of Biomedical Engineering, Indian Institute of Technology Ropar, Rupnagar, Punjab, India
[2] Stanford Cardiovascular Institute, Stanford University School of Medicine, Stanford, CA, USA
[3] Department of Pharmacology and Toxicology, National Institute of Pharmaceutical Education and Research, S.A.S. Nagar, Punjab, India
[4] Department of Surgery, McGowan Institute for Regenerative Medicine, University of Pittsburgh School of Medicine, 450 Technology Drive, Pittsburgh, PA, USA
[5] Department of General Surgery, Dayanand Medical College & Hospital, Ludhiana, Punjab, India
[6] Department of Molecular Biology and Biotechnology, Tezpur University, Napaam, Sonitpur, Assam, India

*Corresponding authors: Durba Pal; E-mail: durba.pal@iitrpr.ac.in
Department of Biomedical Engineering, S. S. Bhatnagar Block,
Indian Institute of Technology Ropar,
Rupnagar-140001, Punjab, India
ORCID: 0000-0001-7672-3529, Ph: (+91) 01881-23-2506

Supplemental Figures

Figure S1

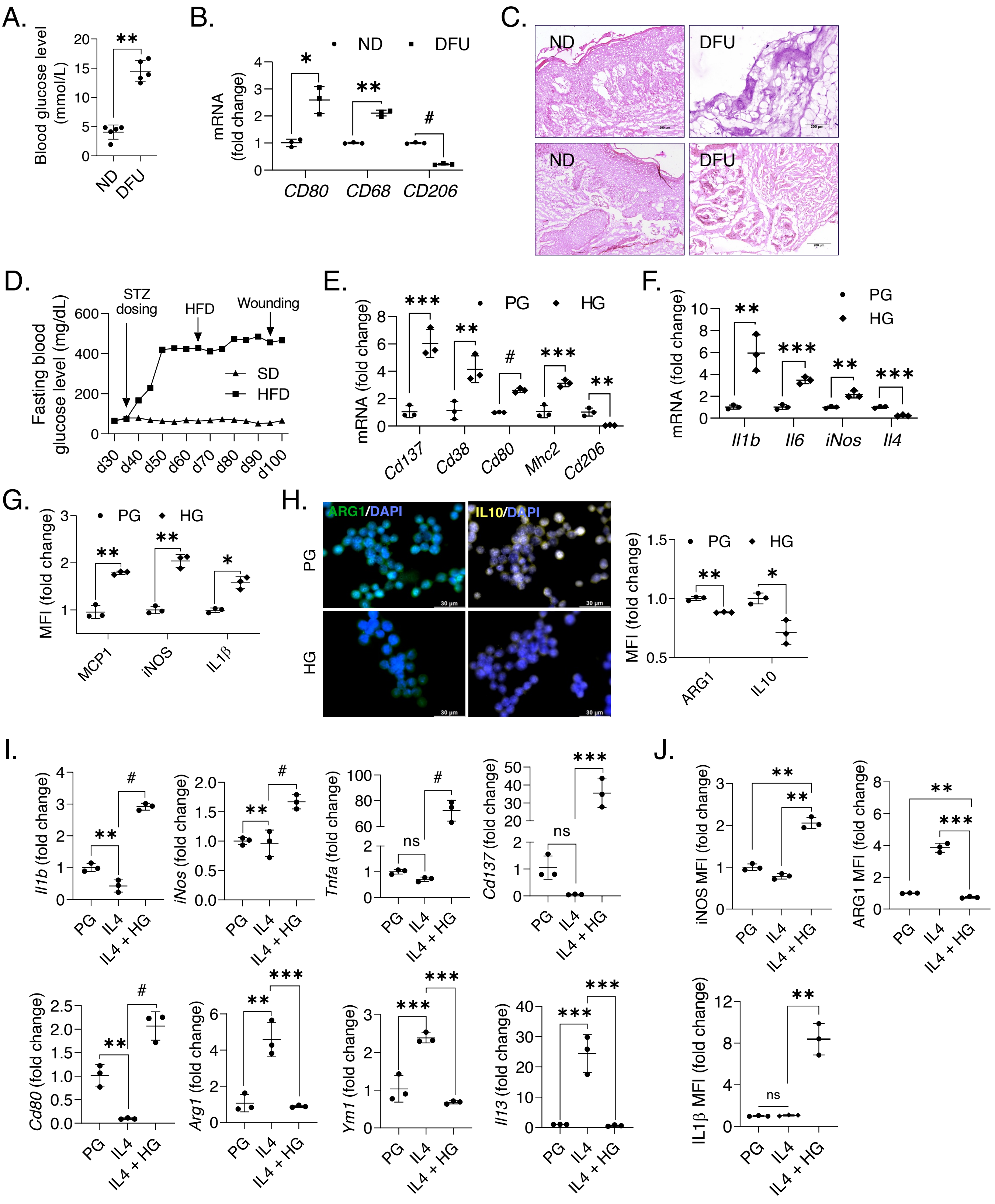

A.
Blood glucose level (mmol/L)
ND
DFU
B.
ND
DFU
mRNA (fold change)
CD80
CD68
CD206
C.
ND
DFU
D.
Fasting blood glucose level (mg/dL)
STZ dosing
HFD
Wounding
SD
HFD
d30
d40
d50
d60
d70
d80
d90
d100
E.
PG
HG
mRNA (fold change)
Cd137
Cd38
Cd80
Mhc2
Cd206
F.
PG
HG
mRNA (fold change)
Il1b
Il6
iNos
Il4
G.
PG
HG
MFI (fold change)
MCP1
iNOS
IL1β
H.
ARG1/DAPI
IL10/DAPI
PG
HG
30 µm
MFI (fold change)
ARG1
IL10
I.
Il1b (fold change)
iNos (fold change)
Tnfa (fold change)
Cd137 (fold change)
Cd80 (fold change)
Arg1 (fold change)
Ym1 (fold change)
Il13 (fold change)
PG
IL4
IL4 + HG
ns
J.
iNOS MFI (fold change)
ARG1 MFI (fold change)
IL1β MFI (fold change)

Figure S1. (**A**) Fasting blood glucose levels are elevated in diabetic subjects compared to non-diabetic individuals. (**B**) Gene expression assessment demonstrates significant overexpression of pro-inflammatory mediators in wound-resident macrophages of diabetic patients. (**C**) Histological analysis of wound areas of non-diabetic and diabetic patients' wound tissue samples (scale 200 μm; n=3/group). (**D**) Fasting blood glucose level of SD and HFD mice (n=3/group). mRNA expression profile of (**E**) cellular markers and (**F**) cytokines in PG and HG treated murine macrophages. (**G**) Measurement of fluorescence intensity for MCP1, iNOS, and IL1β in PG and HG treated macrophages (scale 30 μm; n=3). (**H**) Representative Immunofluorescence images and analysis of ARG1 and IL10 expression in HG (25 mM D-glucose) treated RAW264.7 cells, (scale 30 μm; n=3). (**I**) Relative mRNA expression of inflammatory markers in PG, IL4, IL4 + HG treated RAW264.7 cells. (**J**) Measurement of fluorescence intensity for iNOS, ARG1, and IL1β in PG, IL4, and IL4+HG treated macrophages. Data are expressed as means ± SD; **P*<0.05, ***P*<0.01, ****P*<0.001, $^{\#}$*P*<0.0001 were considered significant difference and ns indicates non-significant. ND, non-diabetic; DFU, diabetic foot ulcer; SD, standard diet; HFD, high fat diet; PG, physiological glucose level, HG, hyperglycemia.

Figure S2

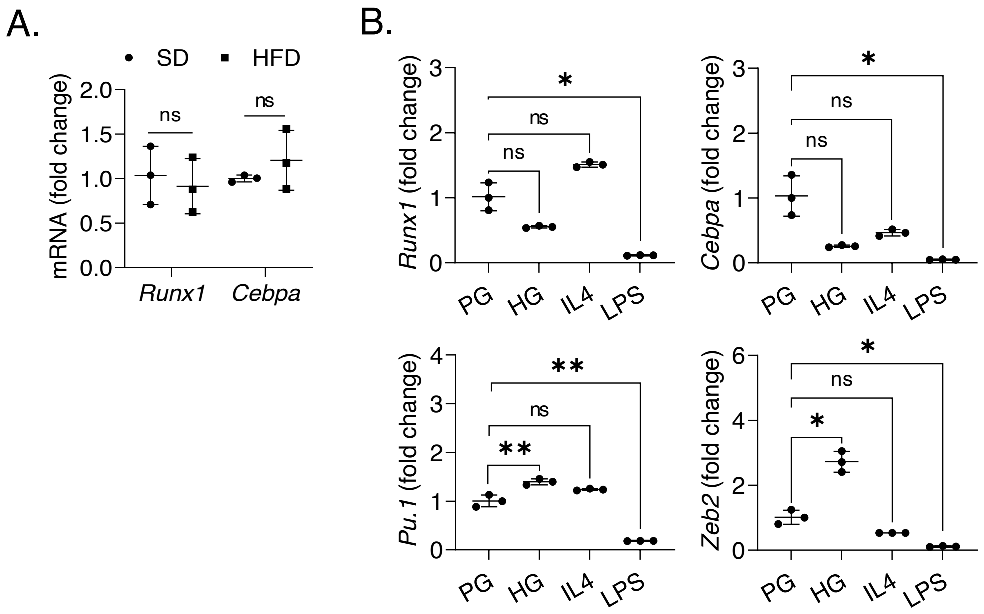

Figure S2. (**A**) Relative mRNA expression of macrophage specific markers in SD and HFD fed mice wound tissue macrophages. (**B**) Relative mRNA expression of macrophage specific markers in PG, HG, IL4, LPS treated RAW264.7 cells. Data are expressed as means ± SD; **P*<0.05, ***P*<0.01, ****P*<0.001, #*P*<0.0001 were considered significant difference and ns indicates non-significant. ND, non-diabetic; DFU, diabetic foot ulcer; SD, standard diet; HFD, high fat diet; PG, physiological glucose level, HG, hyperglycemia.

Figure S3

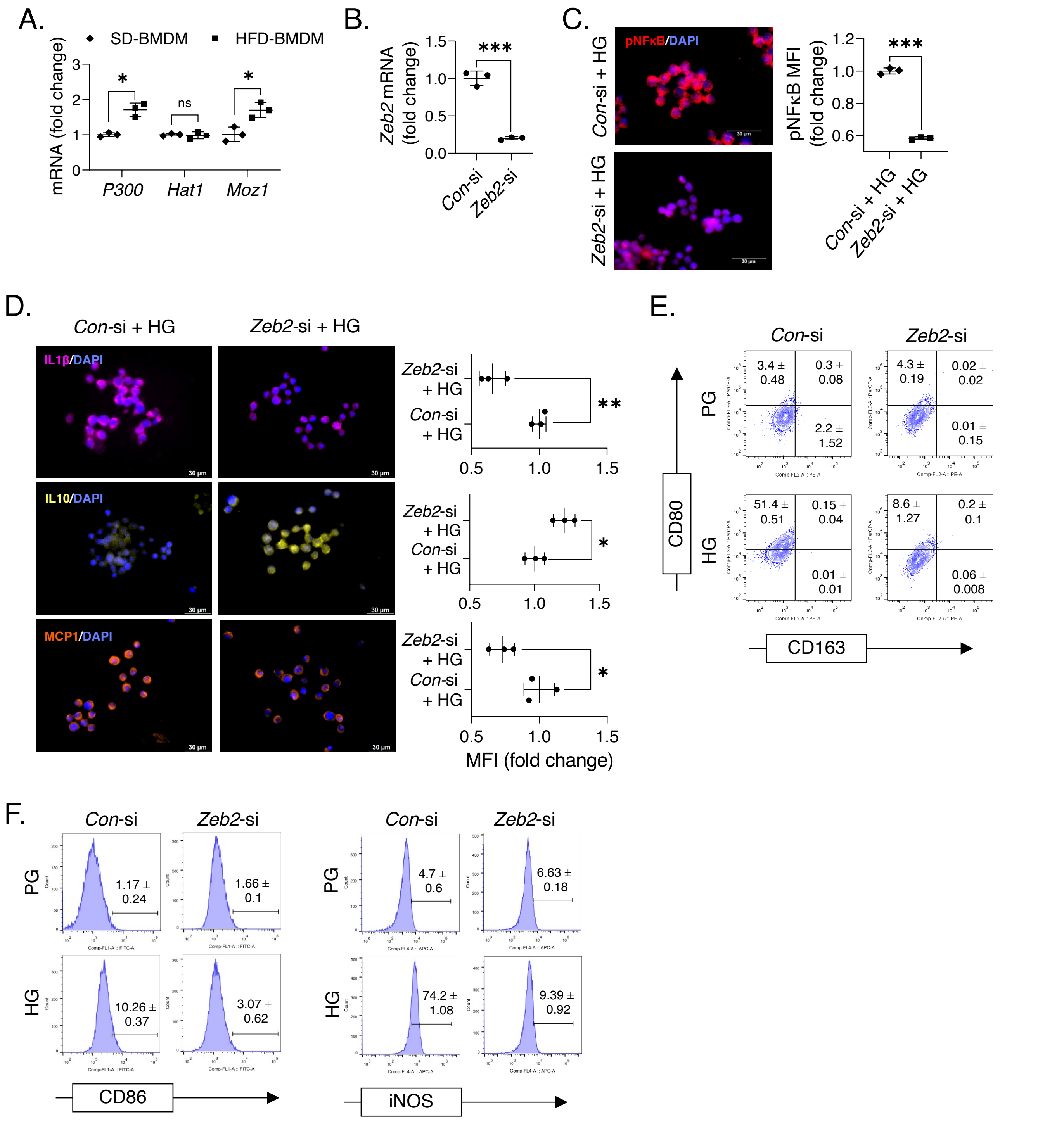

Figure S3. (**A**) mRNA expression profile of acetylation enzyme-related protein in BMDM (Bone Marrow Derived Macrophage) isolated from SD and HFD fed mice. (**B**) *Zeb2* mRNA expression profile in RAW264.7 macrophages after transfecting *Zeb2*-siRNA. (**C**) Immunofluorescence images and analysis of pNFκB in *Zeb2* silenced HG cells (scale 30 µm; n=3). (**D**) Expression of inflammatory cytokines in *Con*-si and *Zeb2*-si transfected HG RAW264.7 cells (scale 30 µm; n=3). Flow cytometry analysis of cell surface markers (**E**) CD80, CD163, and (**F**) CD86, iNOS in *Con*-si and *Zeb2*-si transfected PG and HG cells. Data are expressed as means ± SD; **P*<0.05, ***P*<0.01, ****P*<0.001, #*P*<0.0001 were considered significant difference and ns indicates non-significant. SD, standard diet; HFD, high fat diet; PG, physiological glucose level, HG, hyperglycemia.

Figure S4

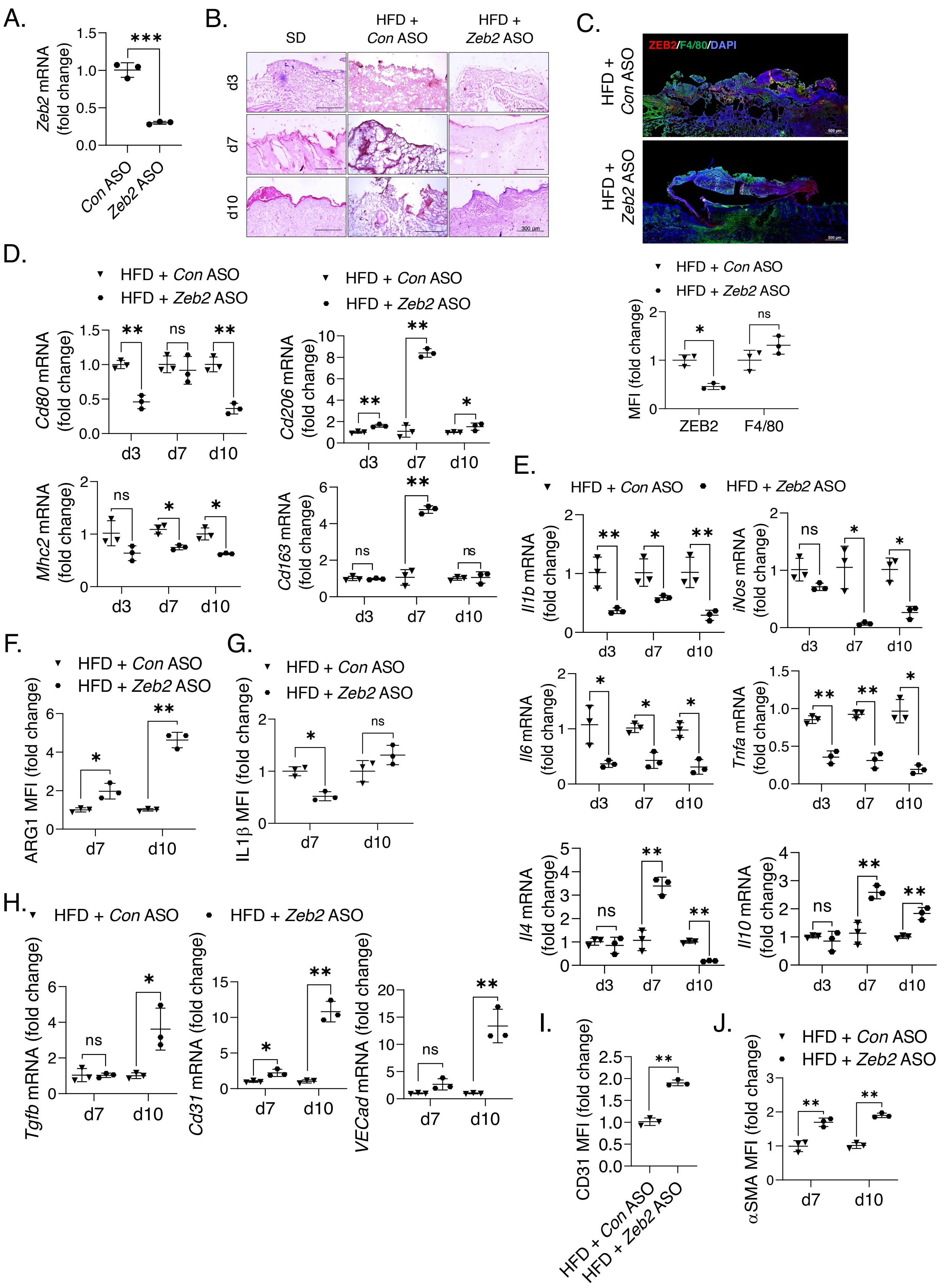

A.
Zeb2 mRNA (fold change)
***
Con ASO
Zeb2 ASO
B.
SD
HFD + Con ASO
HFD + Zeb2 ASO
d3
d7
d10
300 µm
C.
HFD + Con ASO
HFD + Zeb2 ASO
ZEB2/F4/80/DAPI
500 µm
HFD + Con ASO
HFD + Zeb2 ASO
MFI (fold change)
ZEB2
F4/80
D.
HFD + Con ASO
HFD + Zeb2 ASO
Cd80 mRNA (fold change)
Cd206 mRNA (fold change)
Mhc2 mRNA (fold change)
Cd163 mRNA (fold change)
d3
d7
d10
E.
HFD + Con ASO
HFD + Zeb2 ASO
Il1b mRNA (fold change)
iNos mRNA (fold change)
Il6 mRNA (fold change)
Tnfa mRNA (fold change)
Il4 mRNA (fold change)
Il10 mRNA (fold change)
F.
HFD + Con ASO
HFD + Zeb2 ASO
ARG1 MFI (fold change)
G.
HFD + Con ASO
HFD + Zeb2 ASO
IL1β MFI (fold change)
H.
HFD + Con ASO
HFD + Zeb2 ASO
Tgfb mRNA (fold change)
Cd31 mRNA (fold change)
VECad mRNA (fold change)
I.
CD31 MFI (fold change)
HFD + Con ASO
HFD + Zeb2 ASO
J.
HFD + Con ASO
HFD + Zeb2 ASO
αSMA MFI (fold change)

Figure S4. (**A**) *Zeb2* mRNA expression profile in RAW264.7 macrophages after transfecting *Zeb2*-ASO. (**B**) H&E images of wounds tissues at different time point in SD, HFD + *Con* ASO, and HFD + *Zeb2* ASO groups (scale 1000 μm, n=3/group). (**C**) Immunostaining of mice wound tissue by ZEB2 (red), and F4/80 (green) antibody in HFD group treated with or without *Zeb2* ASO, (scale 500 μm, n=3/group). Relative mRNA expression of (**D**) pro and anti-inflammatory cell surface markers, (**E**) pro and anti-inflammatory cytokines at different time point of wound tissue from HFD + *Con* ASO, and HFD + *Zeb2* ASO. Measurement of fluorescence intensity for (**F**) ARG1, and (**G**) IL1β in d7 and d10 mice wound tissue (n=3/group). (**H**) Relative mRNA expression of angiogenesis markers at different time point of wound tissue from HFD + *Con* ASO, and HFD + *Zeb2* ASO. Measurement of fluorescence intensity for (**I**) CD31 in d7, and (**J**) αSMA in d7 and d10 mice wound tissue (n=3/group). Data are expressed as means ± SD; **P*<0.05, ***P*<0.01, ****P*<0.001, #*P*<0.0001 were considered significant difference and ns indicates non-significant. SD, standard diet; HFD, high fat diet.

Figure S5

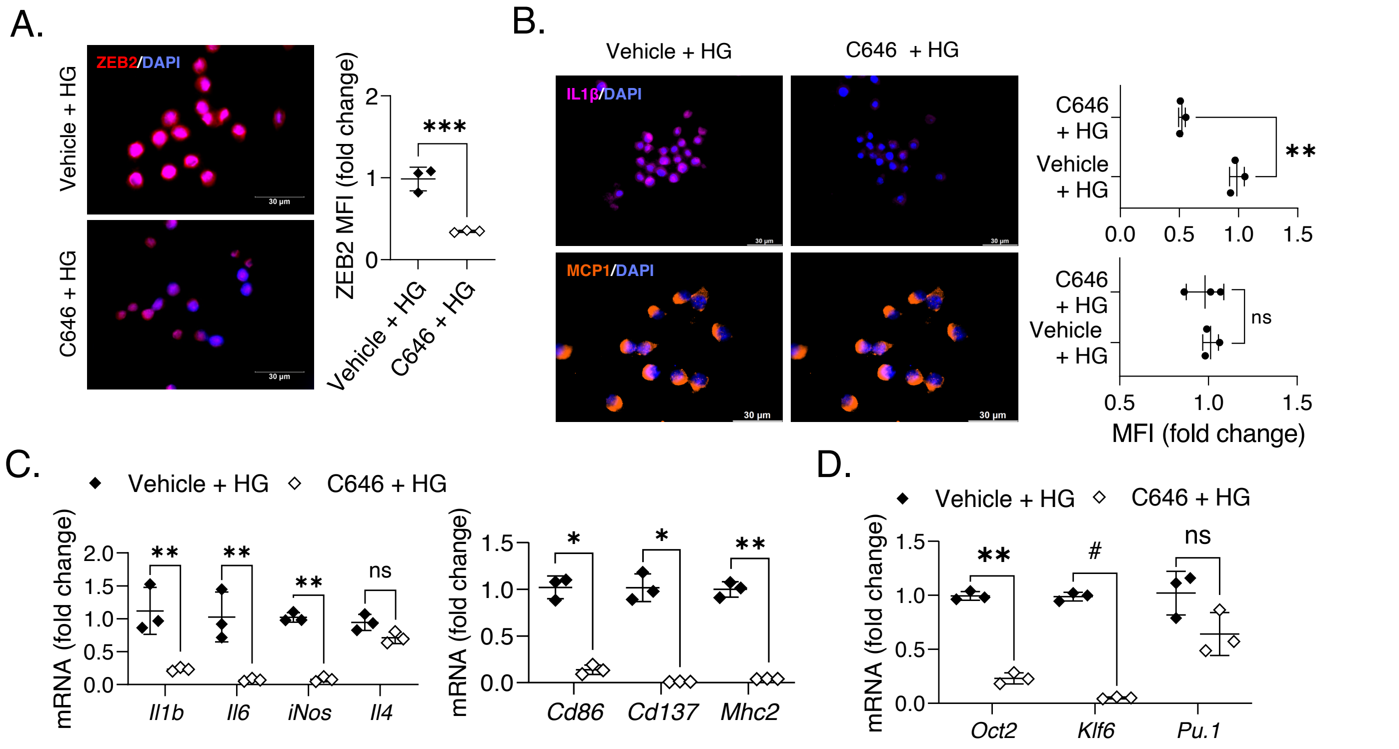

Figure S5. Representative image and analysis of immunostaining for (**A**) ZEB2 and (**B**) pro-inflammatory cytokines in C646 incubated HG treated RAW264.7 (scale 30 µm; n=3). Relative mRNA expression of (**C**) cell surface markers, cytokines, and (**D**) MLDTFs in HG treaded cells incubated with or without C646. Data are expressed as means ± SD; **P*<0.05, ***P*<0.01, ****P*<0.001, #*P*<0.0001 were considered significant difference and ns indicates non-significant. PG, physiological glucose level, HG, hyperglycemia.

Figure S6

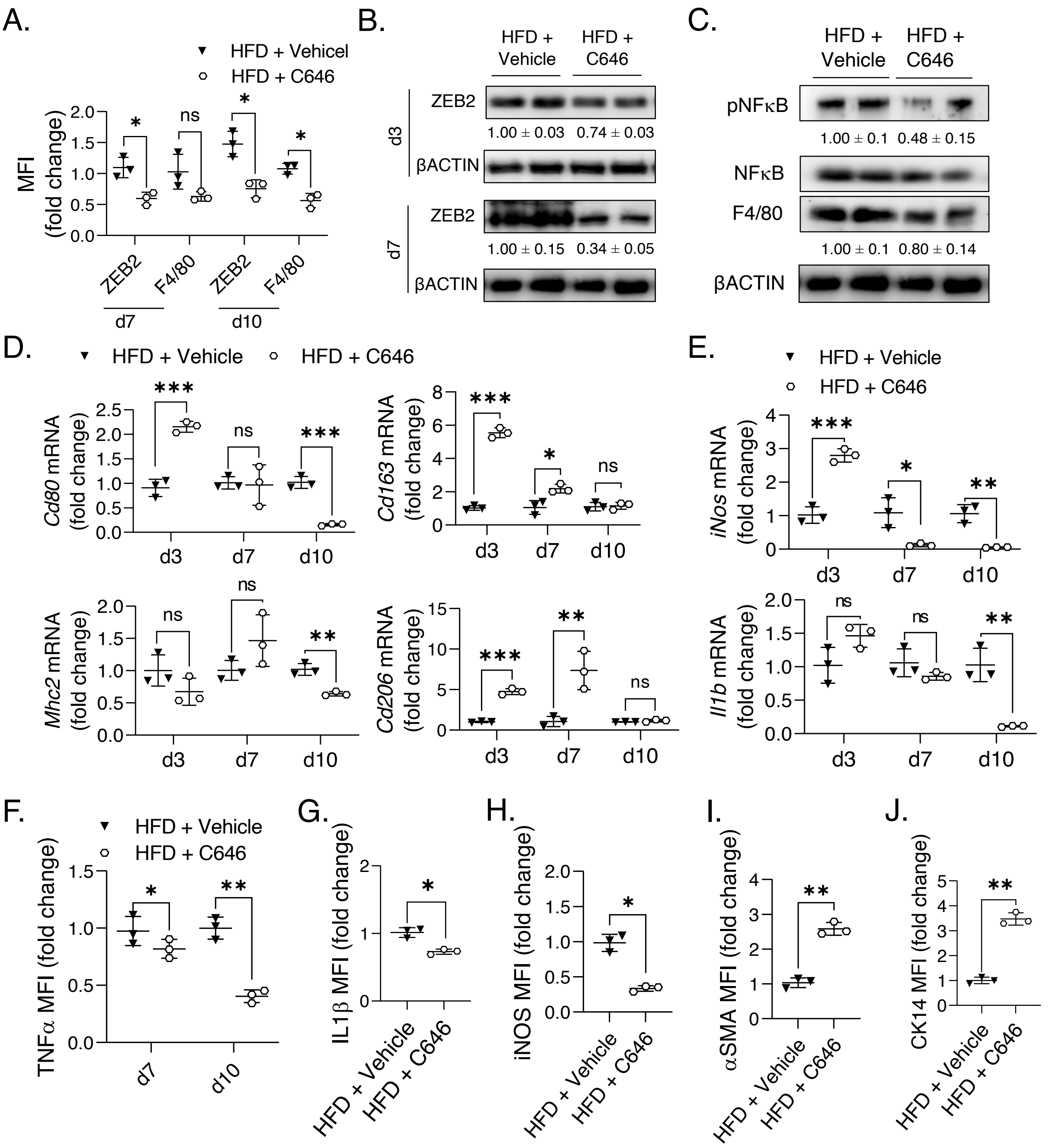

Figure S6. (**A**) Measurement of fluorescence intensity analysis for ZEB2, and F4/80 in d7 and d10 mice wound tissue (n=3/group). Expression of (**B**) ZEB2 at d3, d7 wound tissue, and (**C**) pNFκB, F4/80 at d10 wound tissue of HFD fed mice wound tissue treated with or without C646 (n=4/group). Relative mRNA expression of (**D**) cell surface markers, and (**E**) cytokines at different time points of HFD fed mice wound tissue treated with or without C646. (**F**) Measurement of fluorescence intensity analysis for TNFα in d7 and d10 mice wound tissue (n=3/group). Measurement of fluorescence intensity for (**G**) IL1β in d7, (**H**) iNOS in d10, (**I**) αSMA in d7, and (**J**) CK14 in d10 mice wound tissue. Data are expressed as means ± SD; *P<0.05, **P<0.01, ***P<0.001, #P<0.0001 were considered significant difference and ns indicates non-significant. SD, standard diet; HFD, high fat diet.

Hyperglycemia-induced P300/CBP acetyltransferase drives ZEB2-mediated pro-inflammatory macrophages and delays wound healing

Soumyajit Roy[1], Debarun Patra[1,2], Palla Ramprasad[1], Shivam Sharma[3], Parul Katiyar[4], Ashvind Bawa[5], Kanhaiya Singh[4], Kulbhushan Tikoo[3], Suman Dasgupta[6], Chandan K Sen[4], Durba Pal[1,4*]

[1] Department of Biomedical Engineering, Indian Institute of Technology Ropar, Rupnagar, Punjab, India
[2] Stanford Cardiovascular Institute, Stanford University School of Medicine, Stanford, CA, USA
[3] Department of Pharmacology and Toxicology, National Institute of Pharmaceutical Education and Research, S.A.S. Nagar, Punjab, India
[4] Department of Surgery, McGowan Institute for Regenerative Medicine, University of Pittsburgh School of Medicine, 450 Technology Drive, Pittsburgh, PA, USA
[5] Department of General Surgery, Dayanand Medical College & Hospital, Ludhiana, Punjab, India
[6] Department of Molecular Biology and Biotechnology, Tezpur University, Napaam, Sonitpur, Assam, India

*Corresponding authors: Durba Pal; E-mail: durba.pal@iitrpr.ac.in
Department of Biomedical Engineering, S. S. Bhatnagar Block,
Indian Institute of Technology Ropar,
Rupnagar-140001, Punjab, India
ORCID: 0000-0001-7672-3529, Ph: (+91) 01881-23-2506

Supplementary file

Table A: Patient demographic details

**Pathological features**	
Non-diabetic patients:	
Number of patients	6
Age (median range)	53 ± 6.4
Gender	Male (n = 4), Female (n = 2)
BMI (median range)	22.16 ± 1.91
Fasting serum glucose (mmol/L)	4.0547 ± 1.21
Diabetic patients	
Number of patients	18
Age (median range)	56.75 ± 5.53
Gender	Male (n = 14), Female (n = 4)
BMI (median range)	28.725 ± 5.71
Fasting serum glucose (mmol/L)	11.68624 ± 5.61

Table B: List of antibodies used

Antibody	Dilution	Company	Catalog No.
Phospho-NFκBp65 (S-536)	1:1000 for ICC<br>1:2000 for WB	Abcam	#ab86299
Total NFκB	1:1000 for WB	Invitrogen	#PA1-186
iNOS	1:400 for ICC<br>1:400 for IHC<br>1:1500 for FC	Cell Signaling Technology	#13120
Arginase 1	1:50 for ICC<br>1:400 for IHC	Cell Signaling Technology	#93668
F4/80	1:50 for IHC	Santa Cruz Biotechnology	#sc-377009
CD68	1 µg/mL for IHC	Abcam	#ab125212
βACTIN	1:1000 for WB	Invitrogen	#AM4302
ZEB2	1:100 for IHC	Santa Cruz Biotechnology	#sc-271984
CD80	1 µg/mL for IHC	Abcam	#ab254579
CD163	1 µg/mL for IHC	Abcam	#ab182422
OCT2	1:200 for ICC	Cloud clone	#PAB554Hu01
HAT1	1:200 for ICC	Cloud clone	#PAB952Mu01
P300	1:200 for ICC	Abclonal	#A13016
IL1β	1:200 for ICC	Cell Signaling Technology	#12703T
TNFα	1:100 for ICC	Cell Signaling Technology	#11948T
MCP1	1:50 for IHC	Santa Cruz Biotechnology	#sc-52701
Anti-Mouse IgG<br>(Alexa Fluor 488 conjugated)	1:1000 for ICC<br>1:1000 for IHC	Cell Signaling Technology	#4408
Anti-Rabbit IgG<br>(Alexa Fluor 488 conjugated)	5 µg/mL for ICC<br>1:500 for IHC	Invitrogen	#A-11034
HRP conjugated Anti-Mouse<br>IgG antibody	1:20000 for WB	Sigma-Aldrich	#A9044
HRP conjugated Anti-Rabbit<br>IgG antibody	1:20000 for WB	Sigma-Aldrich	#A9169
TruStainFcXTM (anti-mouse CD16/32)	0.1 µg/million<br>cells for FC	BioLegend	#101319
PE/Cy5 anti-mouse CD80	5 µL/ million cells	BioLegend	#104712
FITC anti-mouse CD86	2 µL/ million cells	BioLegend	#105006
APC anti-human CD163	5 µL/million cells<br>for FC	BioLegend	#326510
Acetyl-Histone H3 (Lys27) Antibody	1:20 for ChIP	Cell Signaling Technology	#4353S
Acetyl-Histone H3 (Lys9) (C5B11) Rabbit mAb	1:20 for ChIP	Cell Signaling Technology	#9649S

Table C: Primer sequences used

**Mouse primers**		
**Gene**	**Forward (5'-3')**	**Reverse (5'-3')**
Cd163	TGCTCAGGAAACCAATCCCA	ACCTCCACTCTTCCAGCG
Cd206	TTCAGCTATTGGACGCGAGG	GAATCTGACACCCAGCGGAA
Cd86	CTGTAGGCAGCACGGACTTG	CATGGTGCATCTGGGGTCCAT
Mhc2	GAAGACGACATTGAGGCCGA	GGAACACAGTCGCTTGAGGA
Il4	GCATGGCCCAGAAATCAAGG	GAGAAATCGATGACAGCGCC
iNos	CTTGGTGAAGGGACTGAGCTG	CGTTCTCCGTTCTCTTGCAGT
bActin	GTACTCTGTGTGGATCGGTGG	AGGGTGTAAAACGCAGCTCAG
Il6	GGGACTGATGCTGGTGACAA	ACAGGTCTGTTGGGAGTGGT
Hat 1	TTTCGGTTACAAGGGCCTGA	CAACATCATCTGCCTCCACAC
Moz1	CTGTCCAACCAGCCGCCAA	GCTTCCAGACTCGGGTATCTCC
Pu.1	GCAGGGGATCTGACCAACCT	AGTCATCCGATGGAGGGGC
Zeb2	CCAGAGGAAACAAGGATTTCAG	AGGCCTGACATGTAGTCTTGTG
Oct2	AATGGACCCGACATTAACCA	AAATGGTCGTTTGGCTGAAG
Runx1	CACGCCAGTTCCCTACTCTG	AGGTAGGTGTGGTAGCGAGA
Cebpa	TACCGAGTAGGGGGAGCAAA	TCATTTTTCTCACGGGGCCA
Klf6	AGCCTATCTTGCCGTCCTTT	CGCCTCGGGTTCATTTC
**Human primers**		
**Gene**	**Forward (5'-3')**	**Reverse (5'-3')**
P300	GGCTGTATCAGAGCGTATTGTC	CCTCGAAATAAGGCAATTCC
ZEB2	CCAGAGGAAACAAGGATTTCAG	AGGCCTGACATGTAGTCTTGTG
CD80	CTCTTGGTGCTGGCTGGTCTTT	GCCAGTAGATGCGAGTTTGTGC
CD68	TACATGGCGGTGGAGTACAA	AGGTGGACAGCTGGTGAAAG
OCT2	GCACCACCCACCAAATGTTC	GCCTTCCCTTGAACTCTCCC
CD163	GTAGCGGGAGAGTGGAAGTG	TCCAAATGCGTCCAGAACCT
MOZ1	CTCATCTCCTGTGCCGACTG	TTTGGCATACGGGTGAGTGG
HAT 1	TCGGAAATGGCGGGATTTGG	CGGAACATTGTTGACAGGCT
KLF	AGGATCGAGGCTTGTGATGC	GTAGCCCAAAAATGCCCACC
βACTIN	ACAGAGCCTCGCCTTTGCC	TCCCAGTTGGTGACGATGC

siRNA and ASO	
Zeb2 siRNA (m)	5'-T*A*A* TACCT*TTG*GGTTCT*C*T*C -3'
Con ASO (non-targeting)	5′- +T*+A*+G*C*C*T*G*T*C*A*C*T*T*+C*+T*+C -3′
Zeb2 ASO (m)	5'-+T*+A*+A* T*A*C*C*T*T*T*G*G*G*T*T*C*T*+C*+T*+C -3'
**siRNA and ASO**	
ZEB2 promoter	FP- TGCGGAGACTTCAAGGTATAATC<br>RP- GACGTGTTACGCCTCTTCTAAT
