# Supplementary material for: Hyperglycemia-induced P300/CBP acetyltransferase drives ZEB2-mediated proinflammatory macrophages and delays wound healing
Source: JCI Insight. 2026 Jan 29;11(5):e192146. doi: 10.1172/jci.insight.192146 (PMC13041667; doi:10.1172/jci.insight.192146)
Supplement: Unedited blot and gel images [file jciinsight-11-192146-s181.pdf]

Hyperglycemia-induced P300/CBP acetyltransferase drives ZEB2-mediated pro-inflammatory macrophages and delays wound healing

Soumyajit Roy[1], Debarun Patra[1,2], Palla Ramprasad[1], Shivam Sharma[3], Parul Katiyar[4], Ashvind Bawa[5], Kanhaiya Singh[4], Kulbhushan Tikoo[3], Suman Dasgupta[6], Chandan K Sen[4], Durba Pal[1,4*]

[1] Department of Biomedical Engineering, Indian Institute of Technology Ropar, Rupnagar, Punjab, India
[2] Stanford Cardiovascular Institute, Stanford University School of Medicine, Stanford, CA, USA
[3] Department of Pharmacology and Toxicology, National Institute of Pharmaceutical Education and Research, S.A.S. Nagar, Punjab, India
[4] Department of Surgery, McGowan Institute for Regenerative Medicine, University of Pittsburgh School of Medicine, 450 Technology Drive, Pittsburgh, PA, USA
[5] Department of General Surgery, Dayanand Medical College & Hospital, Ludhiana, Punjab, India
[6] Department of Molecular Biology and Biotechnology, Tezpur University, Napaam, Sonitpur, Assam, India

*Corresponding authors: Durba Pal; E-mail: durba.pal@iitrpr.ac.in
Department of Biomedical Engineering, S. S. Bhatnagar Block,
Indian Institute of Technology Ropar,
Rupnagar-140001, Punjab, India
ORCID: 0000-0001-7672-3529, Ph: (+91) 01881-23-2506

File for unedited western blot and gel

Full unedited blot for Figure 1.B.

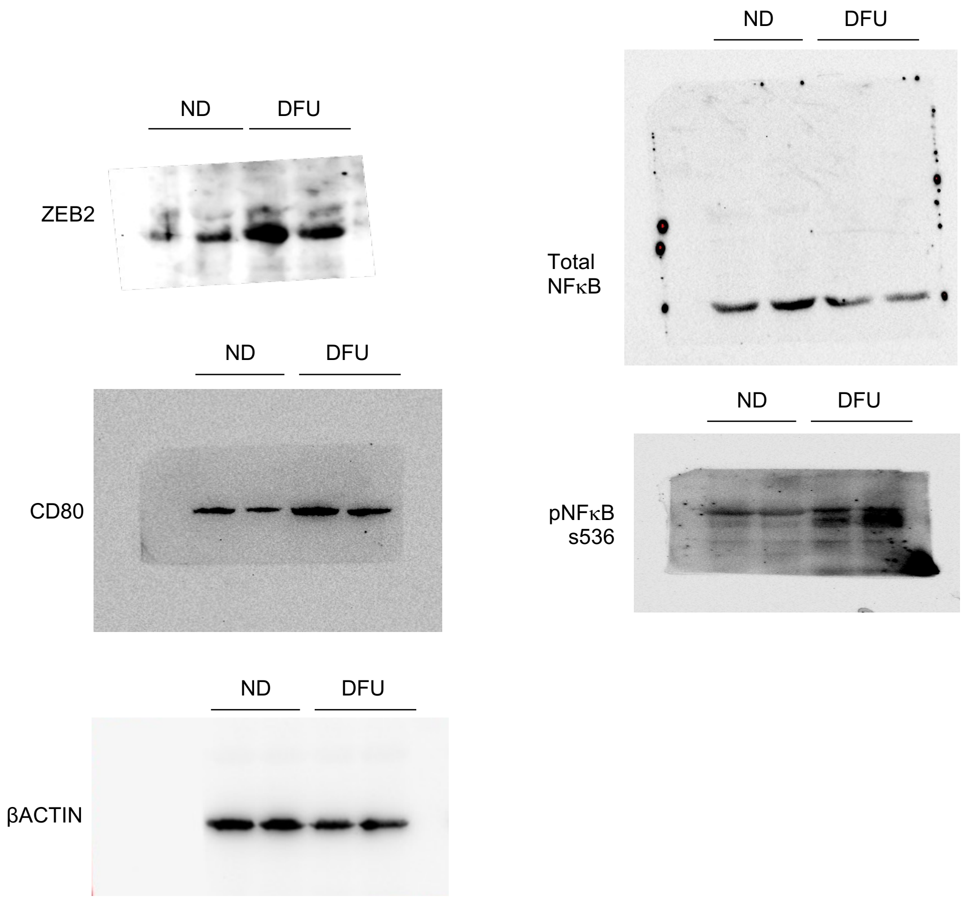

Immunoblotting images of ZEB2, CD80, βACTIN, pNFκB s536, and total NFκB protein in wound tissue macrophages isolated from ND and DFU patients' wound samples. ND, non-diabetic; DFU, diabetic foot ulcer

Full unedited blot for Figure 1.D.

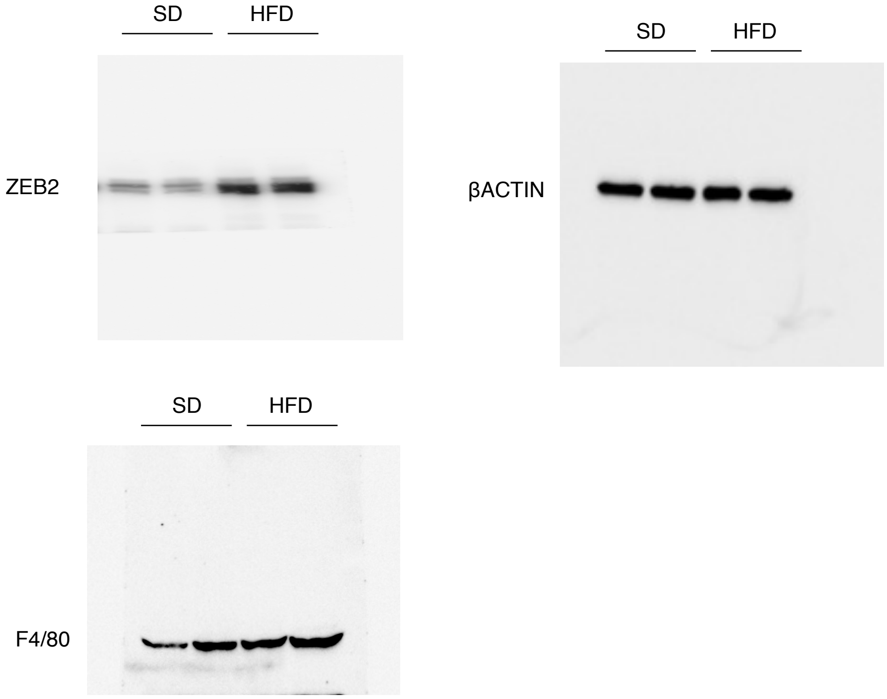

Immunoblotting images of ZEB2, βACTIN, and F4/80 protein in SD and HFD mice wound tissue. SD, standard diet; HFD, high fat diet.

Full unedited blot for Figure 1.E.

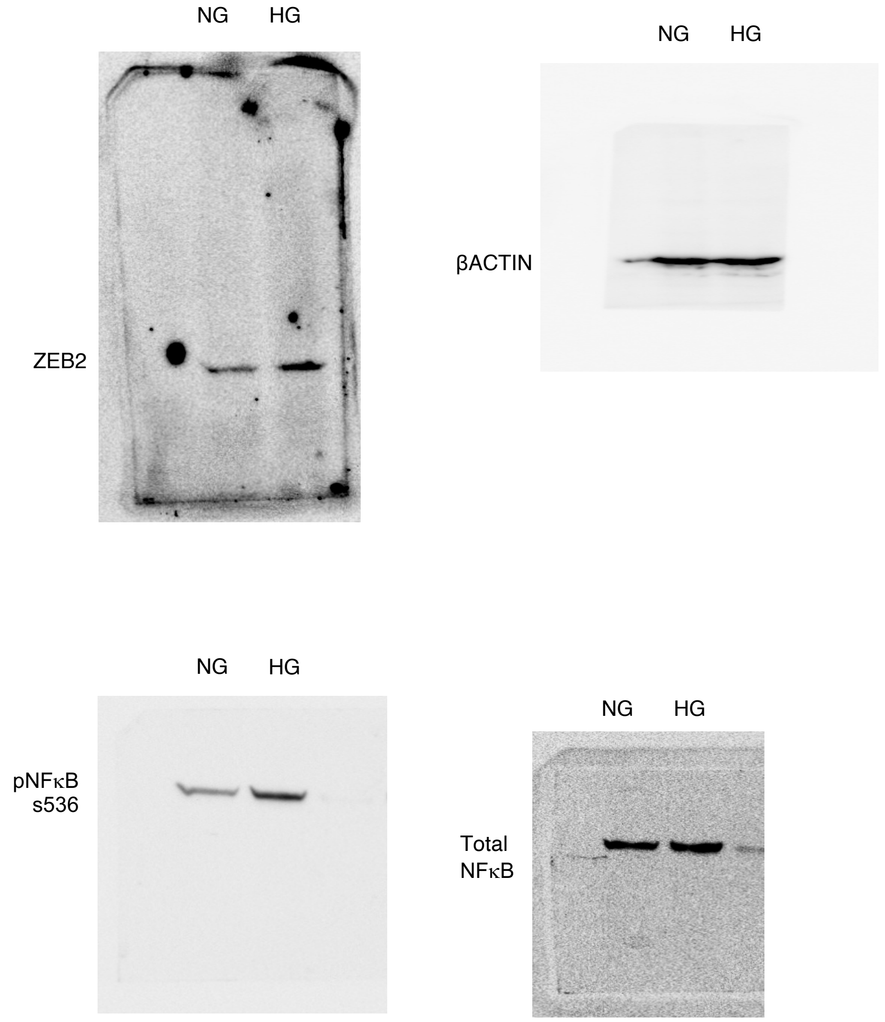

Immunoblotting images of ZEB2, βACTIN, and pNFκB s536, and total NFκB protein in NG and HG treated Raw264.7 macrophages. NG, normoglycemia; HG, hyperglycemia.

Full unedited blot for Figure 3.G.

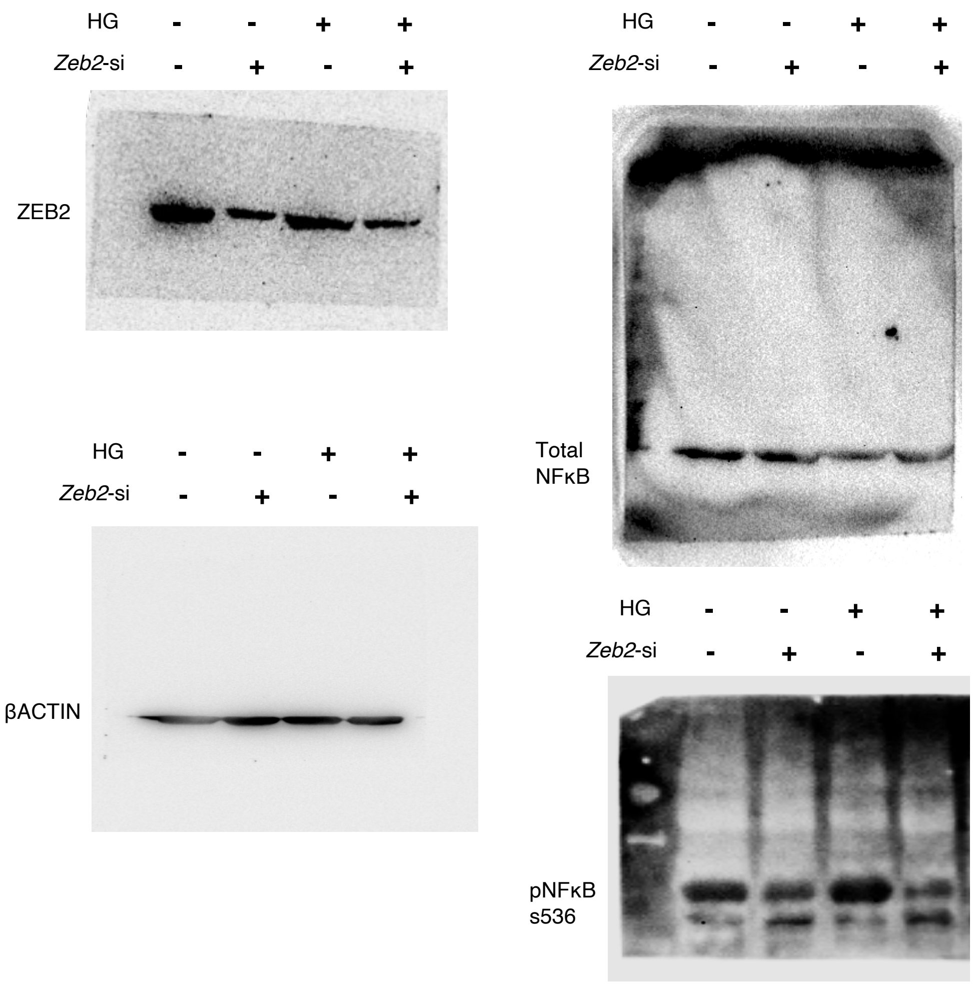

Immunoblotting images of ZEB2, βACTIN, pNFκB s536, and total NFκB protein in NG and HG Raw264.7 macrophages after with/ without *Zeb2*-si treatment. NG, normoglycemia; HG, hyperglycemia.

Full unedited blot for Figure 4.C.

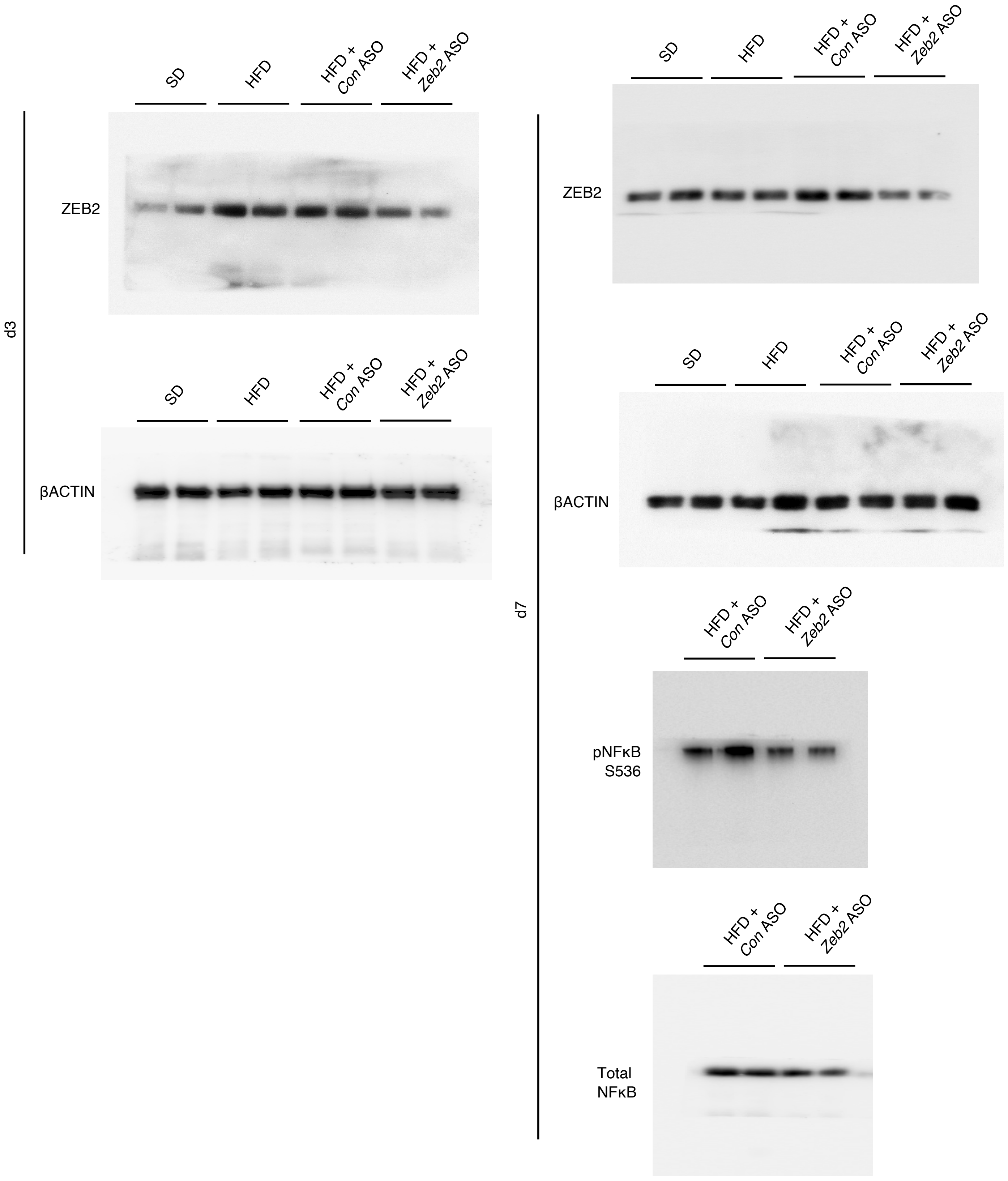

Immunoblotting images of ZEB2, βACTIN, pNFκB s536, and total NFκB protein in SD, HFD, HFD + *Con* ASO, and HFD + *Zeb2* ASO mice wound tissue at d3 and d7. SD, standard diet; HFD, high fat diet.

Full unedited blot for Figure 5.B.

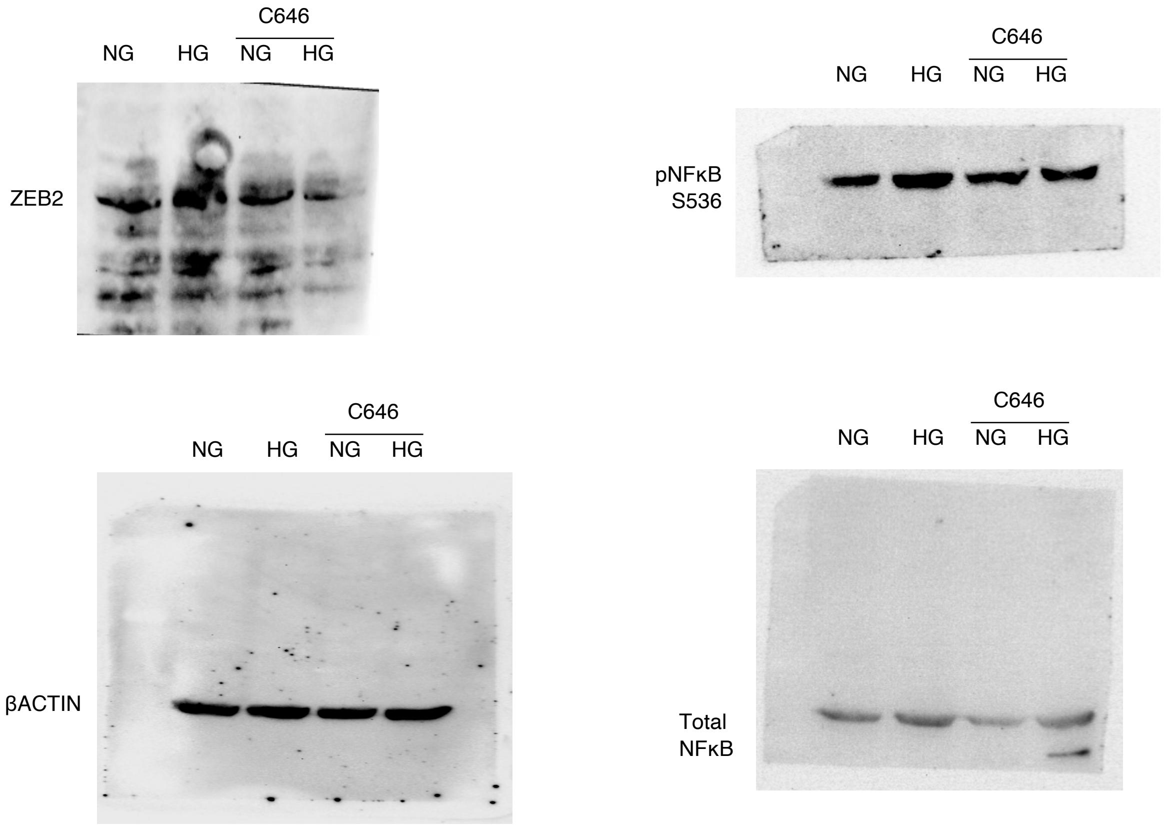

Immunoblotting images of ZEB2, βACTIN, pNFκB s536, and total NFκB protein in NG and HG Raw264.7 macrophages after treating with/without C646. NG, normoglycemia; HG, hyperglycemia.

Full unedited blot for Figure 5.D.

L 1 2 3 4 5 6 7 8

ZEB2 promoter (202bp)

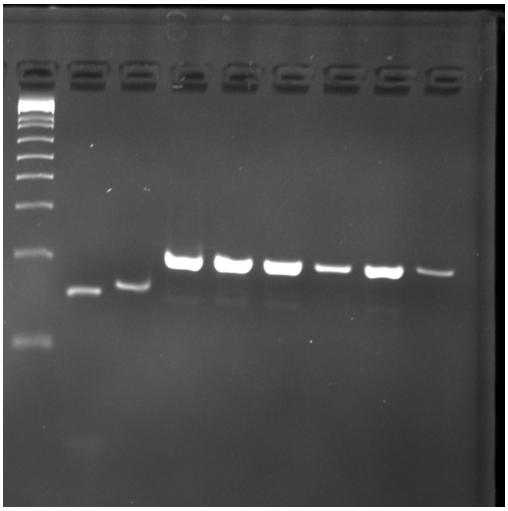

L 1 2 3 4 5 6 7 8

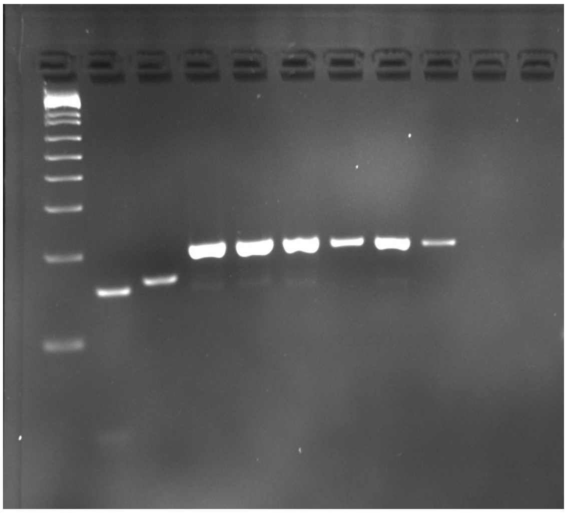

L-1Kb Ladder
1- Negative control
2- Positive control
3- Input NG
4- Input Vehicle + HG
6- Input C646 + HG
5- IP H3K9 NG
7- IP H3K9 Vehicle + HG
8- IP H3K9 C646 + HG

L 1 2 3 4 5 6 7 8

ZEB2 promoter (202bp)

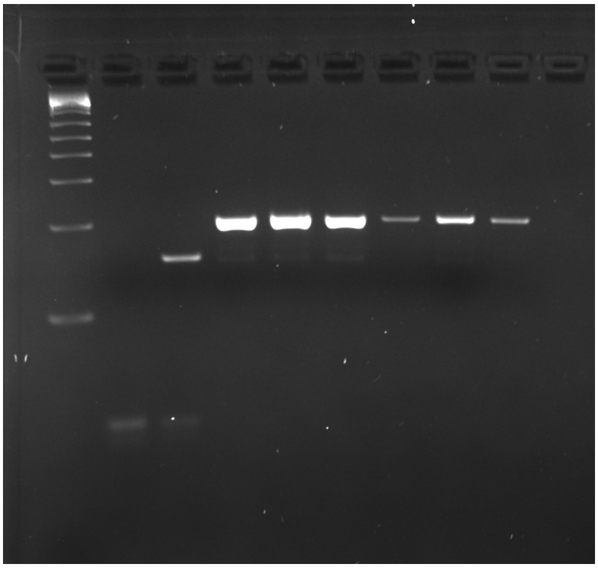

L 1 2 3 4 5 6 7 8

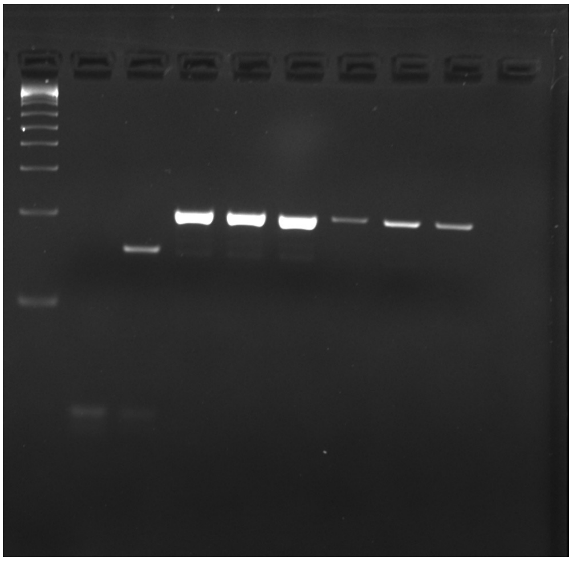

L-1Kb Ladder
1- Negative control
2- Positive control
3- Input NG
4- Input Vehicle + HG
5- Input C646 + HG
6- IP H3K27 NG
7- IP H3K27 Vehicle + HG
8- IP H3K27 C646 + HG

DNA agarose gel electrophoresis of *ZEB2* promoter in input and chromatin immuno-precipitation sample against H3K9Ac and H3K27Ac antibody from NG, HG + Vehicle, C646 + HG group. NG, normoglycemia; HG, hyperglycemia.

Full unedited blot for Figure 5.H.

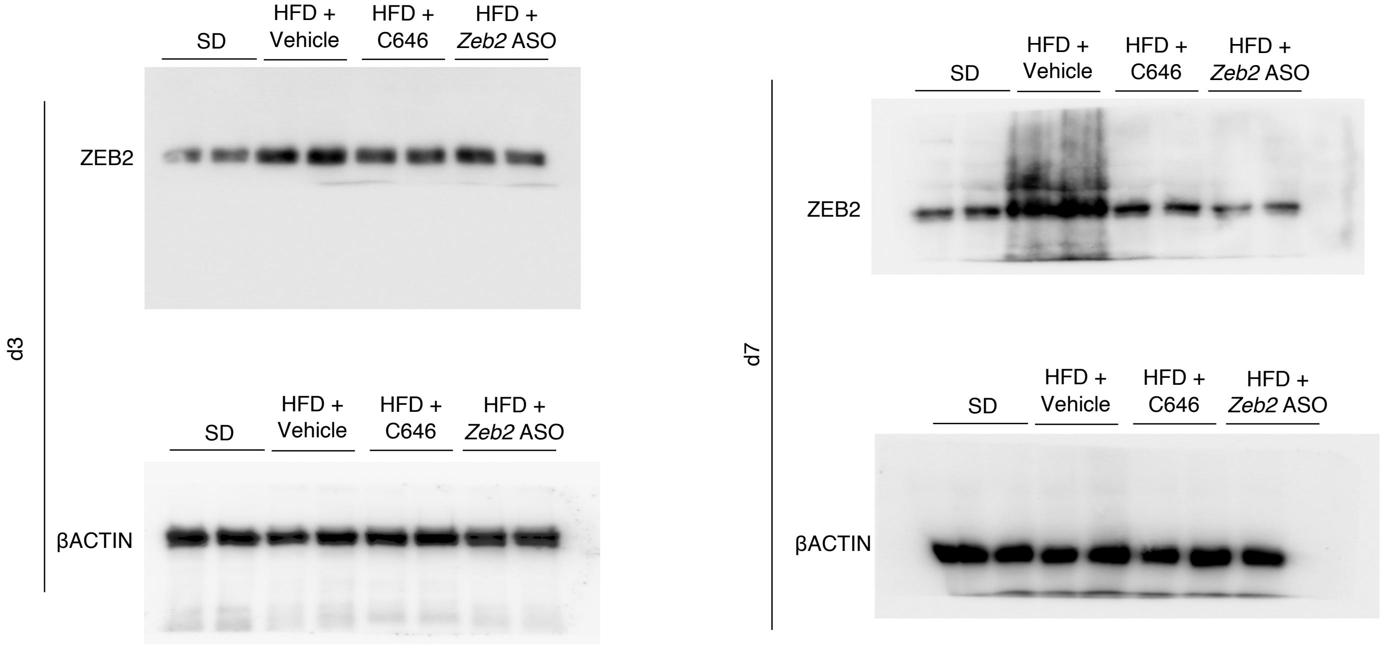

Immunoblotting images of ZEB2, and βACTIN in SD, HFD + Vehicle, HFD + C646, and HFD + *Zeb2* ASO mice wound tissue at d3 and d7. SD, standard diet; HFD, high fat diet.

Full unedited blot for Figure S6.F.

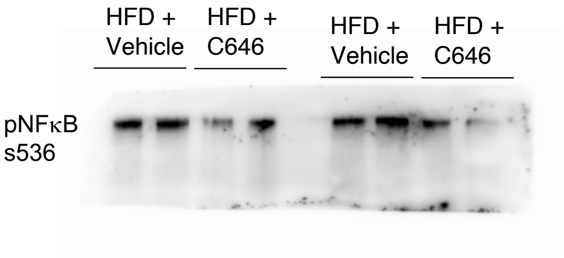

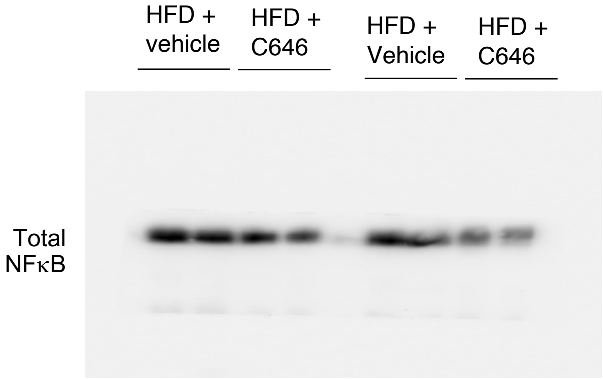

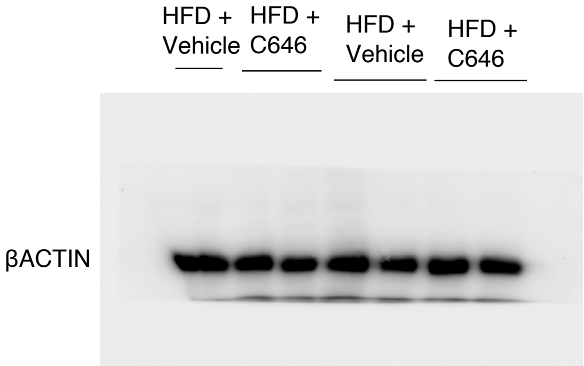

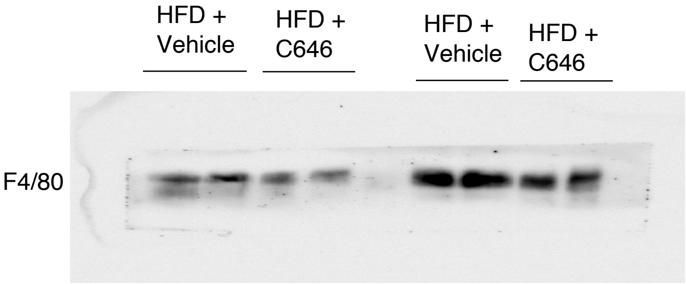

Immunoblotting images of ZEB2, and βACTIN in SD, HFD + Vehicle, HFD + C646, and HFD + *Zeb2* ASO mice wound tissue at d7. SD, standard diet; HFD, high fat diet.
